# Alveolar barrier disruption in varicella pneumonia is associated with neutrophil extracellular trap formation

**DOI:** 10.1172/jci.insight.138900

**Published:** 2020-11-05

**Authors:** Werner J.D. Ouwendijk, Henk-Jan van den Ham, Mark W. Delany, Jeroen J.A. van Kampen, Gijsbert P. van Nierop, Tamana Mehraban, Fatiha Zaaraoui-Boutahar, Wilfred F.J. van IJcken, Judith M.A. van den Brand, Rory D. de Vries, Arno C. Andeweg, Georges M.G.M. Verjans

**Affiliations:** 1 Department of Viroscience, Erasmus MC, Rotterdam, Netherlands.; 2 ENPICOM BV, ‘s-Hertogenbosch, Netherlands.; 3 Department of Pathobiology, Faculty of Veterinary Science, Utrecht University, Utrecht, Netherlands.; 4 Center for Biomics, Erasmus MC, Rotterdam, Netherlands.

**Keywords:** Pulmonology, Virology, Innate immunity, Molecular pathology, Neutrophils

## Abstract

Primary varicella-zoster virus (VZV) infection in adults is often complicated by severe pneumonia, which is difficult to treat and is associated with high morbidity and mortality. Here, the simian varicella virus (SVV) nonhuman primate (NHP) model was used to investigate the pathogenesis of varicella pneumonia. SVV infection resulted in transient fever, viremia, and robust virus replication in alveolar pneumocytes and bronchus-associated lymphoid tissue. Clearance of infectious virus from lungs coincided with robust innate immune responses, leading to recruitment of inflammatory cells, mainly neutrophils and lymphocytes, and finally severe acute lung injury. SVV infection caused neutrophil activation and formation of neutrophil extracellular traps (NETs) in vitro and in vivo. Notably, NETs were also detected in lung and blood specimens of varicella pneumonia patients. Lung pathology in the SVV NHP model was associated with dysregulated expression of alveolar epithelial cell tight junction proteins (claudin-2, claudin-10, and claudin-18) and alveolar endothelial adherens junction protein VE-cadherin. Importantly, factors released by activated neutrophils, including NETs, were sufficient to reduce claudin-18 and VE-cadherin expression in NHP lung slice cultures. Collectively, the data indicate that alveolar barrier disruption in varicella pneumonia is associated with NET formation.

## Abstract 2

A key role for neutrophil activation and impaired alveolar epithelial-endothelial barrier integrity is identified in the pathogenesis of varicella pneumonia.

## Introduction

Most adults worldwide are infected with the human alphaherpesvirus varicella-zoster virus (VZV) (1). VZV causes varicella (chickenpox) as a primary infection, establishes a lifelong latent infection in ganglionic neurons, and reactivates in one-third of latently infected individuals to cause herpes zoster (shingles) (2). While primary VZV infection is generally benign in children, varicella is more severe and frequently accompanied by complications in adults and immunocompromised individuals (2). Pneumonia is the most common and serious complication of chickenpox in adults, with an incidence of 1.0 to 2.3 cases per 400 varicella patients (3, 4). Furthermore, mild pneumonitis may be even more prevalent as radiographic abnormalities are detected in 16% of adult varicella patients (5). Although prompt administration of antivirals strongly reduces mortality rates, about 20% of patients succumb to the disease due to secondary bacterial infections or acute respiratory distress syndrome (ARDS) (6).

The pathogenesis of varicella pneumonia is largely unknown, mainly because VZV is a human-restricted pathogen that does not cause disease in experimental animal models (7, 8) and due to the limited availability of clinical specimens from VZV pneumonia patients. VZV is transmitted via aerosols and presumably gains access to the body via infection of epithelial cells in the upper respiratory tract (9, 10). Although some virus may also reach the lower respiratory tract directly, VZV pneumonia typically develops after onset of skin rash about 2 weeks after infection (2, 11). Histological examination of affected lung tissue shows disseminated interstitial pneumonitis, focal hemorrhagic necrosis, fibrinous exudate in the alveolar spaces, and inflammatory cells in the alveolar wall and air sacs (11, 12). Notably, similar pathological findings are observed in nonhuman primates experimentally inoculated with simian varicella virus (SVV) (13, 14), the causative agent of varicella and herpes zoster in Old World monkeys (8). SVV and VZV genomes are colinear and share about 75% DNA homology (15). More importantly, SVV infection of nonhuman primates recapitulates most if not all pathological, virological, and immunological features of VZV infection in humans (8, 16–18).

There is an unmet need to determine the virus and host factors involved in the pathogenesis of VZV pneumonia. Elucidation thereof will potentially lead to the identification of novel biomarkers that more accurately predict disease severity and guide the development of more effective intervention strategies. In this study, we used the experimental SVV nonhuman primate model to unravel the cell types and mechanisms involved in the pathogenesis of varicella pneumonia.

## Results

### SVV infection causes fulminant varicella pneumonia.

**Figure 1:**
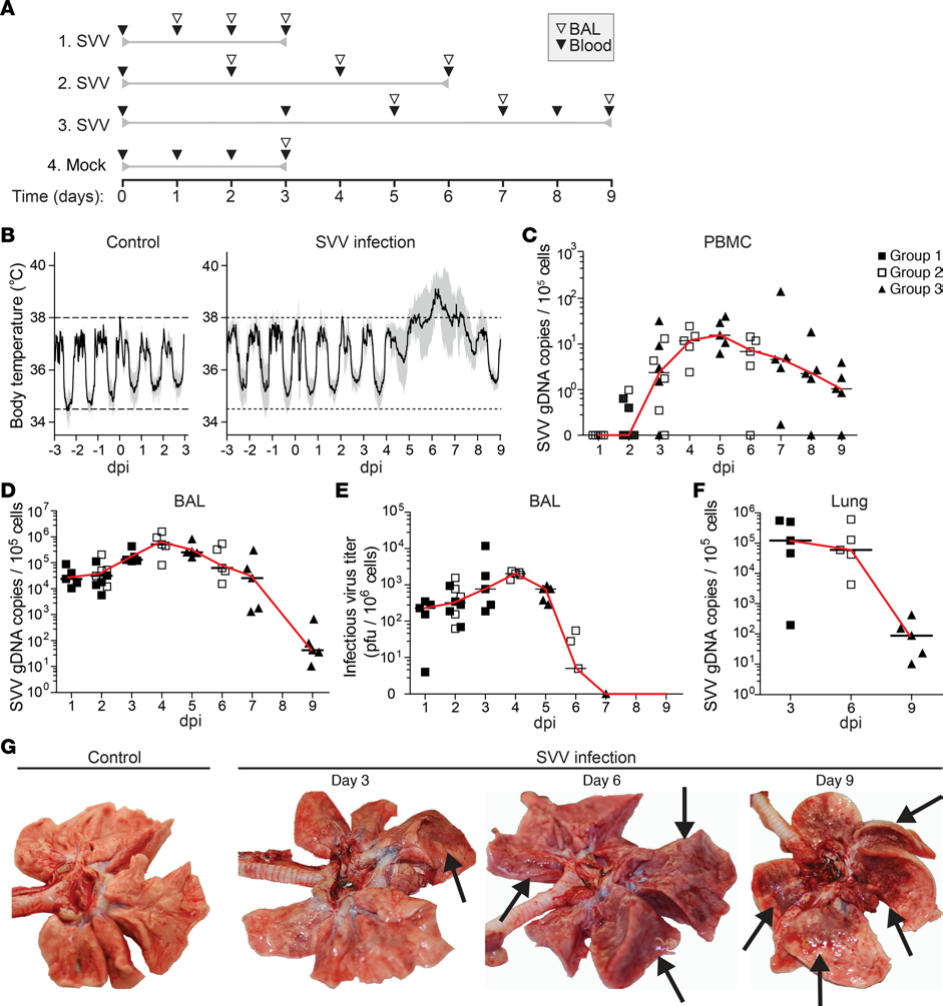
SVV infection of cynomolgus macaques causes transient fever and fulminant pneumonia. (**A**) Experimental design. Time relative to mock or SVV infection (day 0) is indicated. Sampling time points and type are indicated by open (BAL) and closed (blood) inverted triangles. (**B**) Fluctuations in body temperature after mock (control) and SVV infection. Horizontal dashed lines, normal range in body temperature. Black solid line, mean. Gray filled area, 95% CI. Control, *n* = 5; SVV infection, *n* = 15 for 1–3 dpi, *n* = 10 for 4–6 dpi, and *n* = 5 for 7–9 dpi. (**C**, **D**, and **F**) SVV DNA qPCR on PBMC (**C**), BAL cells (**D**) and lung tissue (**F**). Data are expressed as SVV genomic DNA (gDNA) copies per 1 × 10^5^ cells. (**E**) Infectious SVV titers in BAL cells. (**C–F**) Symbols indicate values of individual animals (group 1, filled square; group 2, open square; group 3, filled triangle); black horizontal line indicates median. (**G**) Photographs of lungs from control and SVV-infected animals. Arrows indicate lesions.

Fifteen juvenile cynomolgus macaques were intratracheally infected with SVV and euthanized at 3 (*n* = 5; group 1), 6 (*n* = 5; group 2), and 9 (*n* = 5; group 3) days postinfection (dpi) (Figure 1A). Five control animals were mock infected and euthanized at 3 dpi. SVV infection caused fever from 5 to 8 dpi (Figure 1B). Viral DNA was first detected in blood at 2 dpi (in 3 of 10 animals) and peaked at 5 dpi (Figure 1C). All SVV-infected animals became dyspneic after 6 dpi, indicative of varicella severe pneumonia. SVV DNA load in bronchoalveolar lavage (BAL) cells peaked at 4 dpi and declined slowly thereafter (Figure 1D). By contrast, recovery of infectious SVV peaked at 4 dpi and abruptly decreased after 5 dpi (Figure 1E). SVV DNA loads in lung tissue were comparable with those in BAL cells (Figure 1F). Lungs of SVV-infected animals showed multifocal mild pulmonary consolidation with mild emphysema at 3 dpi. Lungs showed moderate pulmonary consolidation at 6 dpi, which progressed to multifocal severe lung consolidation and hyperemia (likely interstitial pneumonia) with edema and emphysema at 9 dpi (Figure 1G). Edema was also observed in bronchi at 6 and 9 dpi. No aberrations were observed in control animals. Thus, intratracheal SVV infection of cynomolgus macaques leads to a brisk self-limiting virus replication in the lung, which was associated with clinical signs and gross lung pathology that closely resembled VZV-induced pneumonia in humans (11, 12).

### Varicella pneumonia is associated with severe histopathology in lung tissue.

**Figure 2:**
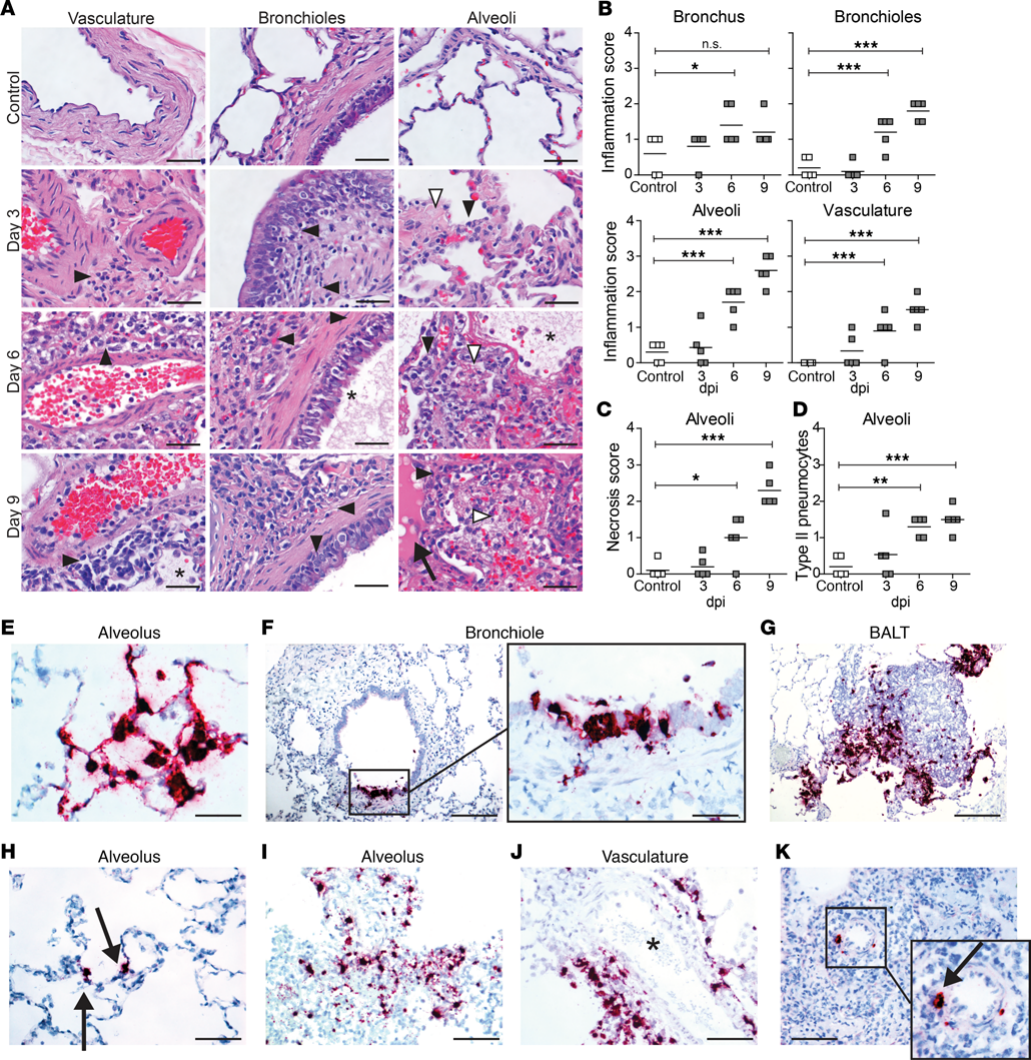
Histopathological changes and detection of SVV ORF63 RNA in lung tissue of SVV-infected cynomolgus macaques. (**A–D**) H&E-stained lung sections (**A**) were scored for inflammation (**B**), necrosis (**C**), and abundance of type II pneumocytes (**D**). *P < 0.05, **P < 0.01, ***P < 0.001 by 1-way ANOVA and Bonferroni’s correction. (**A**) Black arrowheads, inflammatory cells; white arrowheads, necrosis; asterisks, fibrin deposition; arrow, edema. Scale bar: 50 μm. (**E–K**) Lung sections stained for SVV *ORF63* RNA by ISH (purple) and counterstained with hematoxylin (blue) at 3 dpi (**E–G**), 6 dpi (**H–J**), and 9 dpi (**K**). Enlargement of areas indicated by black boxes are shown. Scale bars: 50 μm (**E** and **F**, left panel), 100 μm (**H–K**), 200 μm (**F** [right panel] and **G**). Arrows, SVV RNA^+^ cells; asterisk, blood vessel; BALT, bronchus-associated lymphoid tissue.

Primary bronchi of SVV-infected animals showed mild to moderate neutrophilic and lymphoplasmacytic inflammation within the epithelium of primary bronchi at 6 and 9 dpi (Figure 2, A and B). Foci of epithelial necrosis were present in primary bronchi of SVV-infected, but not in mock-infected, animals (Supplemental Figure 1, A and B; supplemental material available online with this article; https://doi.org/10.1172/jci.insight.138900DS1). SVV infection was associated with pronounced inflammation within bronchiolar walls, alveolar walls and alveolar lumen, and perivascular infiltrates (Figure 2, A and B). Bronchioles showed lymphoplasmacytic inflammation with focal hyperplasia of mucosal epithelium at 3 dpi, which progressed in severity at 6 dpi and 9 dpi (Figure 2, A and B). Lymphoplasmacytic inflammation, necrosis, fibrin deposition, and edema fluid were observed in alveoli of SVV-infected animals at 6 dpi. At 9 dpi, alveolar lumina showed severe lymphoplasmacytic inflammation, necrosis, hemorrhage, and fibrin deposition (Figure 2, A–C). Alveolar damage was associated with increase of type II pneumocytes (Figure 2D), most likely representing tissue regeneration. Thus, intratracheal SVV infection of cynomolgus macaques leads to severe histopathology in the lower respiratory tract at 6 dpi — and especially at 9 dpi.

### SVV replicates in epithelial cells and lymphocytes in lung tissue.

Lung sections were analyzed for presence of SVV gene *ORF63* RNA, one of the most abundantly expressed viral transcripts during lytic infection (19), by in situ hybridization (ISH). SVV-infected cells were readily detected and widely disseminated throughout the analyzed tissue sections at 3 dpi (Figure 2, E–G, and Supplemental Figure 1C). SVV predominantly infected alveolar epithelial cells (Figure 2E), in some animals associated with SVV-infected inflammatory cell infiltrates. To a lesser extent, SVV infected epithelial cells of terminal bronchi and bronchioles, as well as cells within the bronchus-associated lymphoid tissue (BALT) (Figure 2, F and G). At 6 dpi, the number of SVV-infected cells was lower compared with 3 dpi and more variable between animals. While occasional SVV-infected alveolar epithelial cells could be observed, SVV ORF63 transcription was predominantly observed in cells scattered throughout alveolar inflammatory cell infiltrates (Figure 2, H and I). Frequently, SVV-infected cells were localized in close proximity to blood vessels (Figure 2J), and occasionally virus-infected cells were detected in BALT. By contrast, SVV-infected cells were rarely detected in lung tissue of SVV-infected animals at 9 dpi. When present, SVV-infected cells localized adjacent to the vasculature (Figure 2K). Thus, consistent with quantitative PCR (qPCR) and virus isolation results (Figure 1), ISH analysis demonstrated that SVV replication in lung peaks early during infection.

### Gene expression profiling in lung tissues from SVV-infected cynomolgus macaques.

To investigate the mechanisms underlying the pathogenesis of varicella pneumonia, we performed microarray gene expression profiling of lung tissues from mock- and SVV-infected animals (Supplemental Figure 2, A and B). Gene set enrichment analysis was performed using the PANTHER enrichment test to identify statistically significant (FDR < 0.05) enriched Gene Ontology (GO) biological processes (20, 21). At 3 dpi, top enriched upregulated pathways in SVV-infected animals were mostly associated with innate antiviral immunity and cell cycle processes, while most significantly decreased pathways were related to neuron/cell development (Supplemental Figure 2C). At 6 dpi, SVV-infected lungs were enriched for upregulated genes involved in both innate and adaptive (antiviral) immune responses and cellular responses to cytokines, with decreased expression of genes involved in developmental processes (Supplemental Figure 2D). Most enriched upregulated pathways at 9 dpi were involved in cell cycle and proliferation, with lowered expression of genes involved in developmental and morphogenesis processes (Supplemental Figure 2E). Overall, these analyses indicate that SVV infection resulted in a rapid induction of innate antiviral responses in virus-infected lung tissue, followed by recruitment and activation of adaptive immune cells and, subsequently, a proliferative response potentially involving the increase of adaptive immune cell populations and/or restoration of lung parenchymal cells at 9 dpi.

### SVV infection induces a robust innate antiviral immune response in lung tissue.

**Figure 3:**
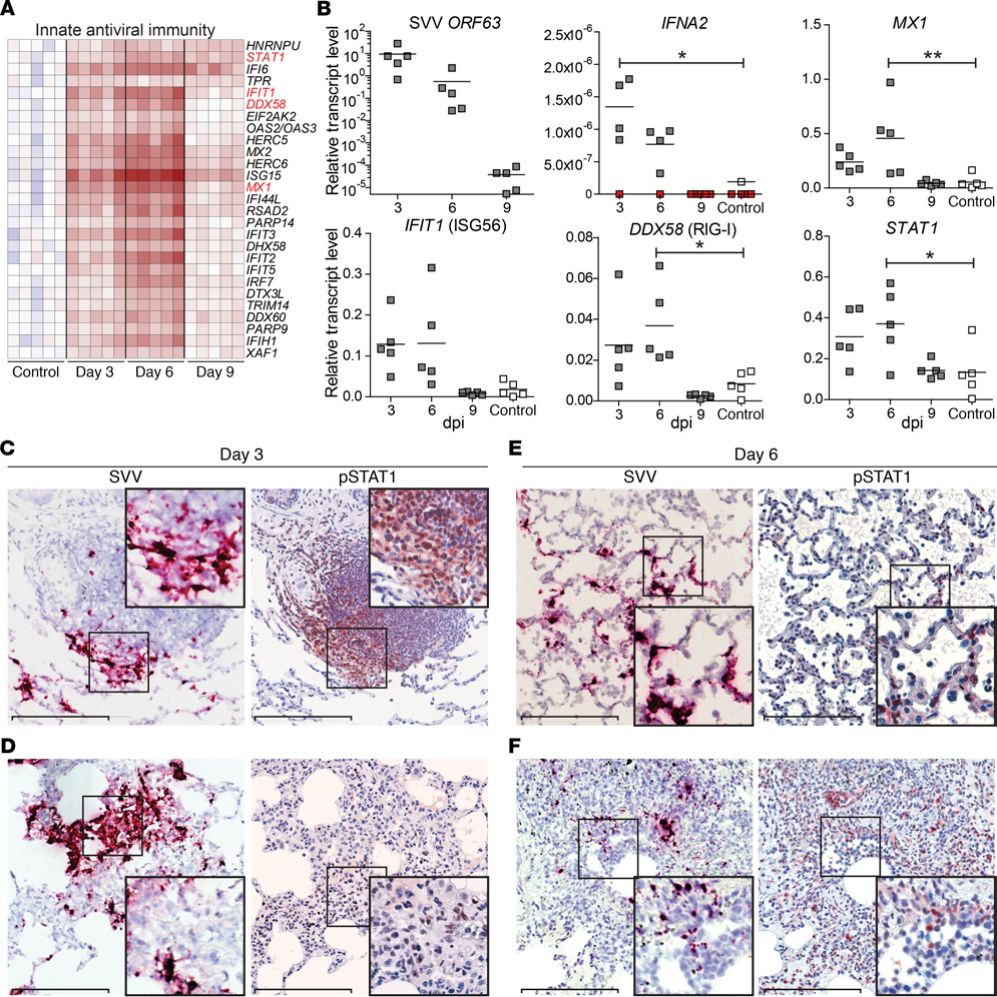
Innate immune responses in lung tissue of SVV-infected cynomolgus macaques. (**A**) Heatmap showing DEGs at 3 dpi involved in innate antiviral immunity. Log_2_-fold change in gene expression is shown. Color gradient: blue, –3; white, 0; red, +6. (**B**) qPCR analysis of the indicated transcripts with data expressed as relative transcript level compared with *GAPDH* using the 2^–ΔCt^ method. Horizontal line indicates mean. Red squares indicate undetectable. *P < 0.05, **P < 0.01 by 1-way ANOVA and Bonferroni’s correction. (**C–F**) Consecutive lung sections stained for SVV *ORF63* RNA (purple) by ISH and pSTAT1 (red) by IHC and counterstained with hematoxylin (blue) at 3 dpi (**C** and **D**) and 6 dpi (**E** and **F**). Enlargement of areas indicated by black boxes are shown. Scale bars: 200 μm.

The prototypic innate antiviral immune response is mediated by type I IFN (IFN-α/β) that inhibit virus replication and recruit inflammatory cells to the site of infection (22). Gene expression analysis revealed that SVV infection resulted in a robust innate antiviral response dominated by IFN-α/β responses at 3 and 6 dpi (Figure 3A). Consistent with ISH and SVV DNA qPCR results (Figures 1E and Figure 2, E–G), SVV ORF63 transcript levels in lung tissue were highest at 3 dpi and steeply declined after 6 dpi (Figure 3B). SVV replication in lung tissue was associated with increased expression of *IFNA2* mRNA (Figure 3B), mainly produced by cells located in alveoli and infiltrating inflammatory cells (Supplemental Figure 3, A and B). Concordant with microarray results, expression of IFN-stimulated genes (ISGs) *MX1*, *IFIT1*, *DDX58*, and *STAT1* was increased at 3 dpi, peaked at 6 dpi, and returned to levels of mock-infected animals at 9 dpi (Figure 3B).

Upon activation of the IFN-α/β receptor, STAT1 is phosphorylated and translocated to the nucleus, leading to transcription of a broad panel of ISGs (22). Therefore, we analyzed the in situ relationship between virus-infected cells and IFN signaling by staining lung sections for SVV *ORF63* RNA and phosphorylated STAT1 (pSTAT1). At 3 dpi, abundant pSTAT1 expression was observed in cells within SVV-infected BALT and occasional inflammatory cell infiltrates juxtaposed to SVV-infected alveolar cells (Figure 3C) — but not in alveolar epithelial cells (Figure 3D). By contrast, more widely distributed and abundant pSTAT1 expression was detected at 6 dpi within alveolar and bronchiolar epithelial cells, as well as inflammatory cell infiltrates located in alveoli and surrounding blood vessels (Figure 3, E and F). Overall, these findings demonstrate that SVV replication in the lung induced a robust IFN-α/β response and STAT1-mediated immune activation of leukocytes in BALT and alveolar infiltrates.

### Clearance of SVV infection in lung tissue is associated with recruitment of inflammatory cells.

**Figure 4:**
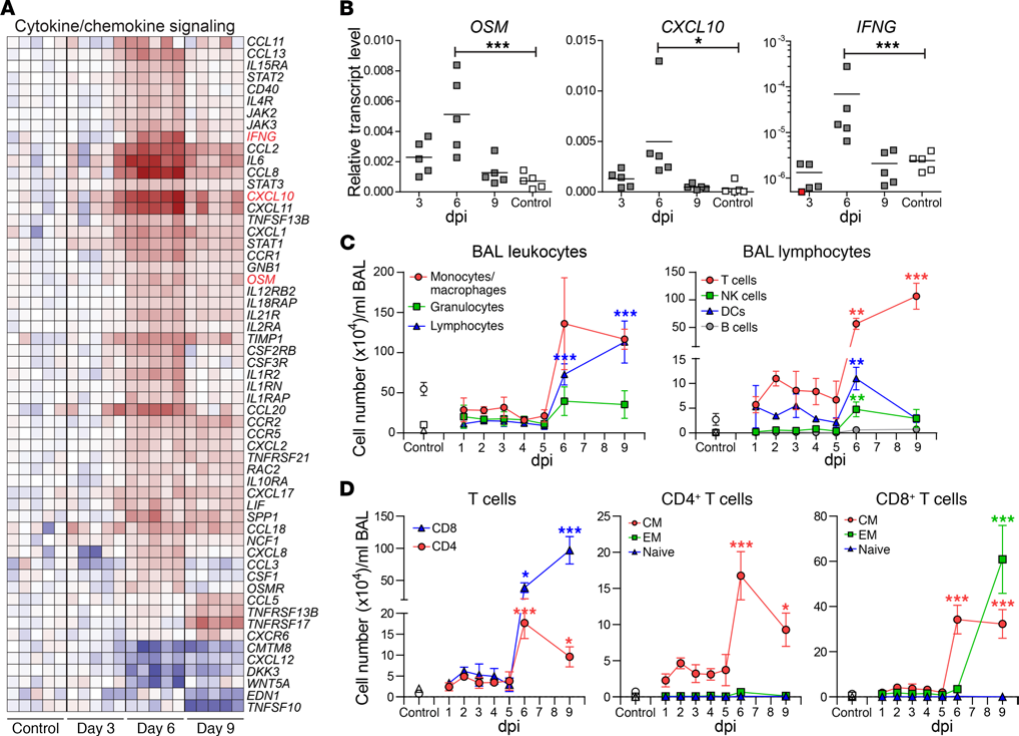
Cytokines, chemokines, and immune cells in BAL and lung tissue of SVV-infected cynomolgus macaques. (**A**) Heatmap showing DEGs involved in cytokine and chemokine signaling. Log_2_-fold change in gene expression is shown. Color gradient: blue, –3; white, 0; red, +6. (**B**) qPCR analysis of the indicated transcripts with data expressed as relative transcript level compared with *GAPDH* using the 2^–ΔCt^ method. Horizontal line indicates mean values. (**C** and **D**) Graphs showing the extrapolated cell number (mean ± SEM) of BAL cell populations (**C**) and T cell populations (**D**), as determined by flow cytometry. CM, central memory; EM, effector memory. *P < 0.05, **P < 0.01, ***P < 0.001 by 1-way ANOVA and Bonferroni’s correction.

To further investigate the mechanisms underlying the inflammatory response in SVV-infected lungs, most notable after cessation of virus replication, the profiles of differentially expressed genes (DEGs) involved in cytokine and chemokine signaling were analyzed (Figure 4A). SVV infection strongly induced expression of IFN-γ and several IL-6 family members (IL-6, leukemia inhibitory factor [*LIF*] and oncostatin-M [*OSM*]) in lung tissue. Chemokines most abundantly induced by SVV infection suggest chemotaxis of neutrophils (*CCL3*, *CXCL1*, *CXCL2*, *CXCL8*, and *SPP1*), monocytes/macrophages (*CCL2*, *CCL8*, *CCL13*, *CXCL10*, *CXCL12*, *CXCL17*, and *SPP1*), and lymphocytes (*CCL2*, *CCL5*, *CCL8*, *CCL13*, *CCL18*, *CCL20*, *CXCL10*, *CXCL11*, and *CXCL17*) at 6 and 9 dpi (Figure 4A). Microarray results were confirmed by qPCR, showing that SVV infection significantly induced *OSM*, *CXCL10*, and *IFNG* mRNA levels at 6 dpi (Figure 4B).

To investigate the kinetics and cell types involved in varicella pneumonia, the phenotype and frequency of cells in BAL samples were analyzed by ex vivo flow cytometry (Supplemental Figure 4, A and B). BAL cells of mock-infected animals were mainly monocytes/macrophages (73% ± 14%; mean ± SD), with lower numbers of granulocytes (14% ± 9%) and lymphocytes (13% ± 8%). SVV infection increased the total number of leukocytes present in BAL samples, especially after 6 dpi (Supplemental Figure 4, C and D), and changed the cell types present in BAL. SVV infection was initially associated with an increase in granulocytes and lymphocytes and a decrease in monocytes/macrophages. From 6 dpi onward, a marked increase in monocytes/macrophage and lymphocytes — and, to a lesser degree, granulocytes — was observed in BAL (Figure 4C and Supplemental Figure 4E). Although serial sampling may influence BAL cell composition, we did not observe any differences between BAL cells obtained at 2 dpi from group 1 (second BAL sample) and group 2 (first BAL sample). The BAL lymphocyte population of mock-infected animals was mainly composed of T cells (79% ± 11%) and NK cells (15% ± 9%) with fewer DCs (5% ± 4%) and B cells (<1%). SVV infection induced a swift increase in the number of BAL DCs, followed by an increase in the NK cells and T cells (Figure 4C), initially central memory CD4^+^ and CD8^+^ T cells, followed by activated effector memory CD8^+^ T cells (Figure 4D and Supplemental Figures 4, F and G).

To determine the spatial distribution of lung-infiltrating inflammatory cell types in SVV-infected animals, lung sections were stained for CD45 (leukocytes), neutrophil elastase (neutrophils), CD68 (monocytes/macrophages), CD3 (T cells), granzyme B (grB; cytotoxic T cells and NK cells), and CD20 (B cells) by IHC. SVV infection caused increased CD45^+^ cell numbers in lung tissue, which colocalized with SVV-infected cells at 3 and 6 dpi (Supplemental Figure 3C). Control animals had low numbers of neutrophils and T cells in their alveolar septa and monocytes/macrophages in the alveolar lumen. The alveoli contained very low numbers of grB^+^ cells and no B cells. SVV infection induced a mild increase in neutrophils, T cells, and grB^+^ cells at 3 dpi (Supplemental Figure 5A). Prominent inflammatory infiltrates composed of neutrophils, T cells, monocytes/macrophages, and grB^+^ cells, but not B cells, were identified in alveoli at 6 dpi and further increased in numbers at 9 dpi (Supplemental Figure 5A). Simultaneously, SVV infection induced a robust expansion of T cell and B cell populations in BALT (Supplemental Figure 5B). The combined flow cytometric and IHC analyses demonstrated that SVV infection resulted in the rapid mobilization of innate immune cells — notably neutrophils, as well as DC and NK cells — to the lung, followed by a more abundant influx of neutrophils, monocytes/macrophages, and CD8^+^ T cells.

**Figure 5:**
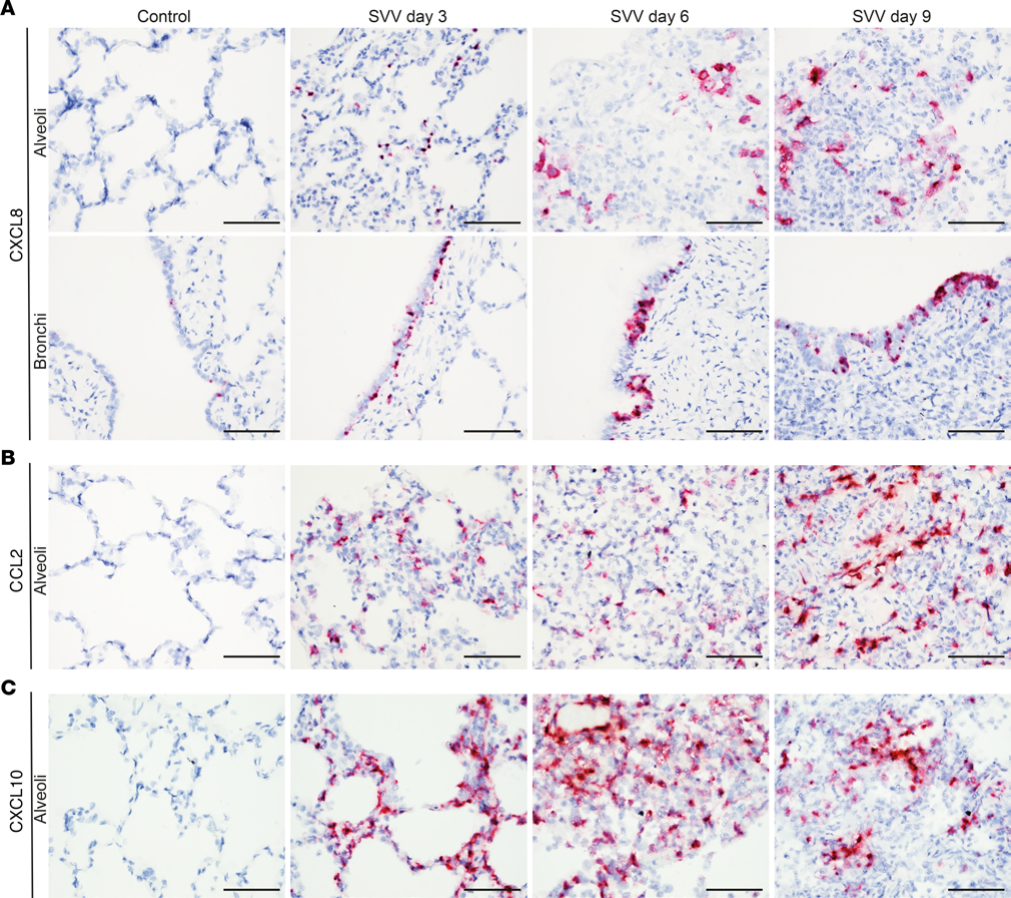
Expression of CCL2, CCL8 and CXCL10 transcripts in lung tissue of SVV-infected cynomolgus macaques. (**A–C**) Lung sections were stained for CXCL8 (**A**), CCL2 (**B**), and CXCL10 (**C**) transcripts by ISH (purple) and counterstained with hematoxylin (blue). Scale bars: 50 μm.

Next, we analyzed the location of cells expressing key chemokines potentially involved in recruitment of neutrophils (*CXCL8*), monocytes/macrophages (*CCL2*), and lymphocytes (*CXCL10*) in lungs of SVV-infected animals. SVV infection induced *CXCL8* expression in bronchiolar epithelial cells and alveolar lining cells at 3 dpi, followed by more abundant expression of *CXCL10* by infiltrating inflammatory at 6 and 9 dpi (Figure 5A). Similarly, *CCL2* and *CXCL10* expression increased over time in the alveoli of SVV-infected animals (Figure 5, B and C). While *CCL2* expression appeared to be restricted to inflammatory cells, *CXCL10* was also abundantly expressed by alveolar epithelial cells (Figure 5, B and C). Thus, the strong IFN-α/β response induced by SVV infection (Figure 3) led to expression of neutrophil-, monocyte/macrophage-, and lymphocyte-recruiting chemokines by lung-resident cells, which was further augmented by the infiltrating inflammatory cells.

### Detection of neutrophils and neutrophil extracellular traps in lung tissue of SVV-infected cynomolgus macaques.

**Figure 6:**
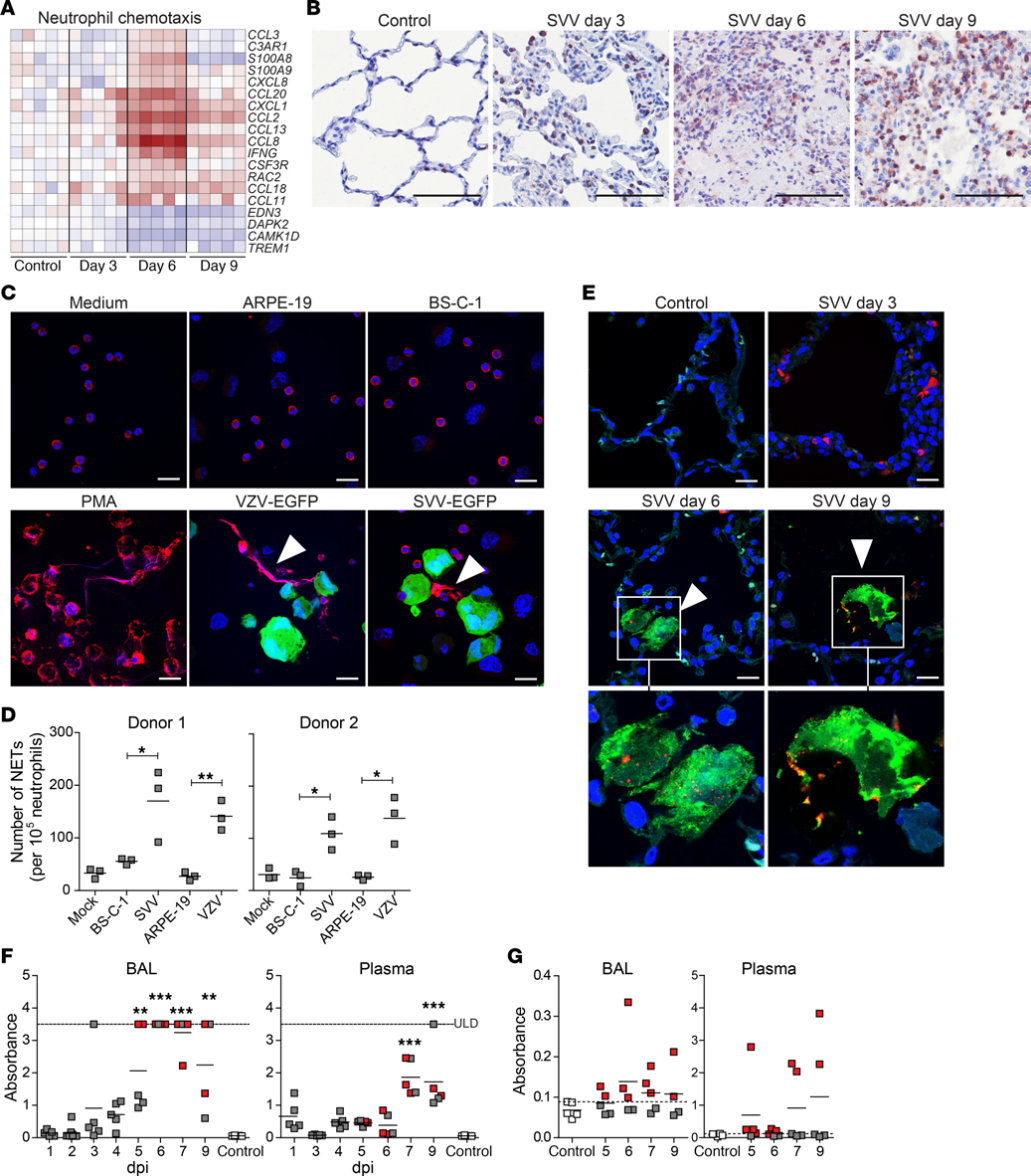
Neutrophils and NETs in lung tissue of SVV-infected cynomolgus macaques. (**A**) Heatmap showing DEGs involved in neutrophil chemotaxis. Log_2_-fold change in gene expression is shown. Color gradient: blue, –3; white, 0; red, +6. (**B**) Lung sections stained for neutrophil elastase by IHC (red) and counterstained with hematoxylin (blue). Scale bar: 100 μm. (**C** and **D**) Human neutrophils were cocultured with medium (mock), PMA, uninfected or SVV-EGFP–infected (green) BS-C-1 cells, and uninfected or VZV-EGFP-infected (green) ARPE-19 cells for 6 hours. NETs were identified by staining for DNA (blue) and neutrophil elastase (red), followed by confocal microscopy (**C**) for quantification (**D**). (**C**) Representative images are shown (*n* = 2 donors, *n* = 3 replicates). Arrowheads indicate NETs. Scale bar: 20 μm. *P < 0.05 and **P < 0.01 by unpaired Student’s *t* test. (**E**) Lung sections stained for citrullinated histone H3 (citH3; green), MPO (red), and DNA (Hoechst-33342; blue). Scale bar: 20 μm. Arrowheads, NETs. Split channel images are shown for the area indicated by white boxes. (**F** and **G**) Detection of His-DNA (**F**) and MPO-DNA (**G**) complexes in BAL and plasma by ELISA. **P < 0.01 and ***P < 0.001 by 1-way ANOVA and Bonferroni’s correction. ULD, upper limit of detection. *y* axis indicates absorbance at 405–492 nm (**F**) and absorbance at 450–620 nm (**G**). (**F** and **G**) Red squares, BAL/plasma samples in which MPO-DNA complexes were detected (absorbance values greater than the maximum absorbance measured in control animals; indicated by dashed line).

Neutrophils are considered key players in the pathogenesis of acute lung injury (ALI), in which accumulation and activation of neutrophils is associated with degranulation and release of proteases, reactive oxygen species, and proinflammatory cytokines (23, 24). Gene expression profiling of lungs of SVV-infected animals showed pronounced upregulation of host genes involved in neutrophil chemotaxis (Figure 6A), consistent with the observed infiltration of neutrophils in lungs of SVV-infected animals (Figure 6B). Moreover, the contribution of neutrophils in combatting pulmonary SVV infection is supported by upregulation of genes involved in neutrophil degranulation (Supplemental Figure 7).

Recent studies implicate an important role for neutrophil extracellular traps (NETs) in ALI due to respiratory bacterial and viral infections (25–28). In response to proinflammatory cytokines, neutrophils can undergo a specialized form of cell death, referred to as NETosis, leading to the release of NETs composed of nuclear chromatin associated with histones and many granular antimicrobial proteins (29, 30). While NETs are considered to physically entrap and neutralize pathogens, excessive NET formation induces alveolar lung damage (26). To analyze whether SVV and VZV can induce NETosis, human neutrophils were exposed to mock- and virus-infected cells. Both viruses significantly induced NET formation in vitro (Figure 6, C and D). Subsequently, lung sections from SVV-infected animals were stained for the NETosis markers lytic granule component myeloperoxidase (MPO) and citrullinated histone H3 (citH3) (31, 32). SVV infection induced the deposition of NETs at 6 and 9 dpi, as demonstrated by detection of DNA meshworks associated with citH3 and MPO expression (Figure 6E). NETs were undetectable at 3 dpi and in control animals. To investigate the kinetics of NET formation, serial BAL samples were analyzed for both cell-free histone-DNA (His-DNA) complexes, a commonly used surrogate marker for NETosis (33, 34), and MPO-DNA complexes, a specific marker for NETs (33, 35). His-DNA complexes were detected at low levels starting from 3 dpi, peaked at 6 dpi, and declined at later times after infection (Figure 6F). Previous studies suggested that His-DNA complexes in plasma correlate with NET formation in the lung (33, 36). Analyses of paired plasma samples showed a similar profile of His-DNA complexes, which peaked at 7 dpi and remained at the same level at 9 dpi (Figure 6F). The presence of NETs was confirmed by detection of MPO-DNA complexes in a subset of BAL and plasma samples (Figure 6G). Notably, MPO-DNA complexes were detected in those animals with highest abundance of His-DNA complexes in BAL and plasma. Collectively, these results suggest an important role for neutrophils and potentially NETosis in acute varicella pneumonia of SVV-infected cynomolgus macaques.

### Detection of NETs in BAL and plasma of severe VZV pneumonia patients.

**Figure 7:**
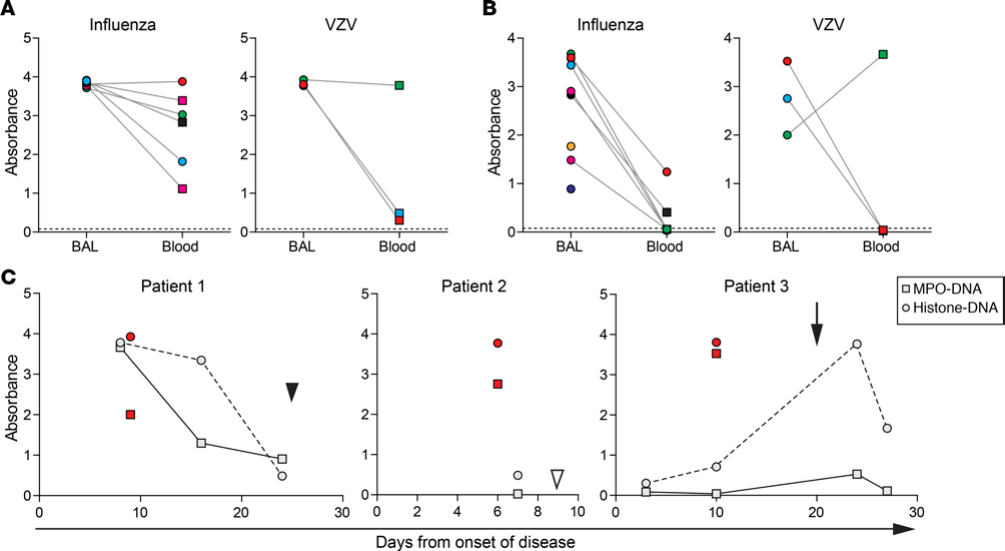
Detection of NETs in patients with severe varicella pneumonia and influenza pneumonia. (**A** and B) Detection of His-DNA (**A**) and MPO-DNA complexes (**B**) in paired BAL and blood samples (obtained ± 2 days of BAL sample) in human influenza and VZV pneumonia patients by ELISA. Squares and circles indicate plasma and serum samples, respectively. Dashed line indicates absorbance in serum of healthy human control subjects. (**C**) Correlation between His-DNA (circles) and MPO-DNA (squares) complexes in BAL (red), paired plasma/serum samples (light gray), and time from onset of disease in VZV pneumonia. Filled arrowhead indicates time of hospital discharge (patient 1); open arrowhead indicates time of death (patient 2); arrow indicates time of secondary bacterial infection of the lung (patient 3). (**A–C**) The *y* axis indicates absorbance at 450–620 nm.

To investigate the potential involvement of NETs in VZV pneumonia, we analyzed surplus BAL and paired (serial) plasma/serum samples, originally obtained for diagnostic purposes, from patients with severe varicella pneumonia (patients 1–3) and influenza pneumonia (patients 4–10) as controls (Supplemental Table 1). Involvement of NETs in influenza pneumonia has recently been described (36). All paired BAL and plasma/serum samples of both the influenza pneumonia and VZV pneumonia patients contained His-DNA complexes (Figure 7A). MPO-DNA complexes were detected in all BAL samples from influenza pneumonia and VZV pneumonia patients (Figure 7B) but only in 2 of 6 available paired plasma/serum samples from influenza cases and 1 of 3 paired plasma samples from VZV cases. Detection of His-DNA and MPO-DNA complexes in blood correlated with disease severity in 1 VZV pneumonia patient (patient 1), with peak NET levels in plasma/serum at 8 days after onset of disease (corresponding to 1 day after admission to the hospital) and steady decline of NET levels until hospital discharge at day 25 (Figure 7C). By contrast, MPO-DNA complexes were undetectable in plasma/serum samples obtained preceding and after NET^+^ BAL samples in patients 2 and 3. Notably, NETs were detected in serum of patient 3 shortly following secondary bacterial infection (*S*. *aureus*) (Figure 7C). Collectively, these findings indicate that NETs are present in BAL, but not necessarily plasma/serum samples, from VZV pneumonia patients.

### Mechanisms of varicella pneumonia–associated ALI.

**Figure 8:**
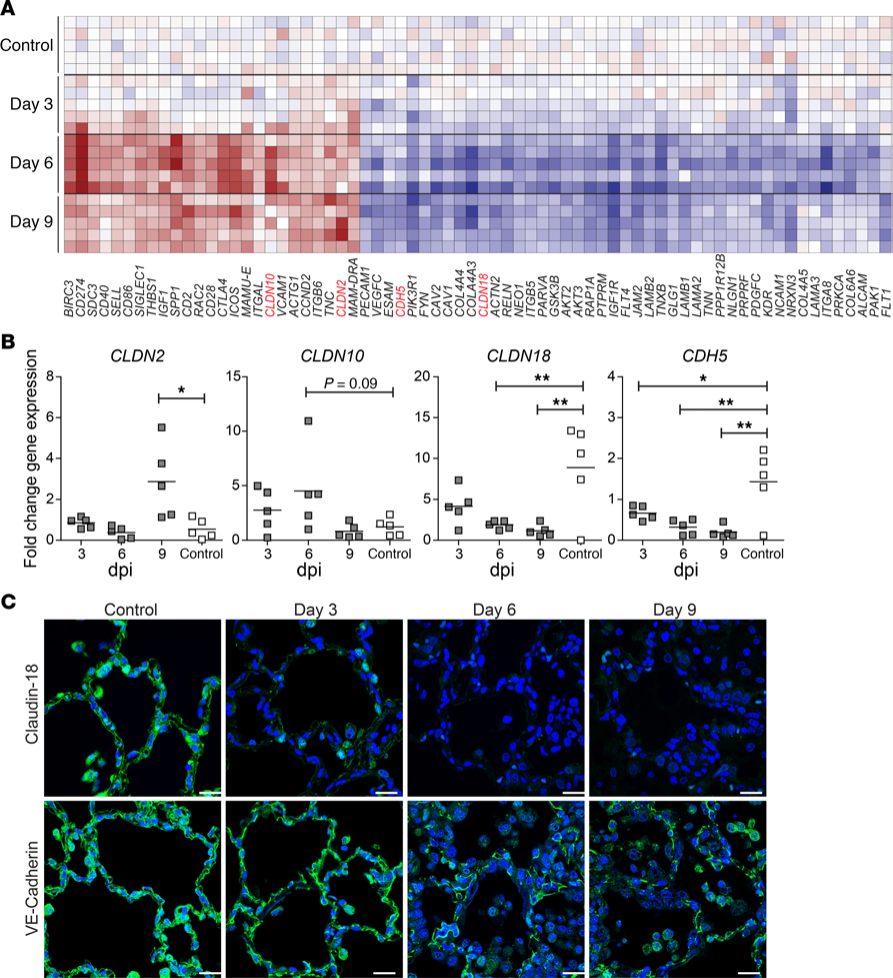
SVV infection affects expression of alveolar epithelial and endothelial cell junction proteins in SVV-infected cynomolgus macaques. (**A**) Heatmap showing DEGs involved in cell adhesion, tight junctions, focal adhesions, and adherens junctions. Log_2_-fold change in gene expression is shown. Color gradient: blue, –3; white, 0; red, +6. Claudin-2 (*CLDN2*), *CLDN10*, *CLDN18*, and VE-cadherin (*CDH5*) are indicated in red. (**B**) qPCR analysis of the indicated transcripts and *GAPDH*. Data are expressed as fold change in gene expression relative to control animals (calibrator) and normalized to *GAPDH* using the 2^–ΔΔCt^ method. Horizontal line indicates mean. *P < 0.05 and **P < 0.01 by 1-way ANOVA and Bonferroni’s correction. (**C**) Lung sections stained for claudin-18 or VE-cadherin (green). Nuclei were stained with Hoechst-33342 (blue). Scale bar: 20 μm.

ALI is associated with the influx of protein-rich edema into the alveoli due to increased permeability of the alveolar epithelial/endothelial-barrier (EEB) (23, 37). Combined fluorescent TUNEL assay and staining for epithelial marker cytokeratin indicated that epithelial cell death is not a major contributor to SVV-induced ALI (Supplemental Figure 6). Next, the expression profiles of DEGs involved in epithelial and endothelial cell-cell junction and cell adhesion molecules in SVV- and mock-infected animals were analyzed (Figure 8A). Notably, SVV infection caused downregulation of claudin-18 (*CLDN18*) and simultaneous upregulation of *CLDN2* and *CLDN10*. Claudin family members are essential for tight junction (TJ) formation and integrity, and they therefore regulate TJ permeability (38, 39). Claudins are divided into 2 classes: sealing claudins (such as claudin-18) and pore-forming claudins (like claudin-2 and claudin-10) that prevent or promote paracellular flux, respectively (38). Additionally, gene expression analysis revealed decreased expression of vascular endothelial cadherin (VE-cadherin, *CDH5*) (Figure 8A), an adherens junction protein that is essential for maintaining the endothelial barrier integrity in lung capillaries (40, 41). Microarray results were confirmed by qPCR, demonstrating that SVV infection significantly induced *CLDN2* and tended to increase *CLDN10* expression, while simultaneously robustly downregulating *CLDN18* and *CDH5* expression in the lung (Figure 8B). Claudin-18 and VE-cadherin proteins were abundantly expressed in lungs of mock-infected animals, which was significantly reduced upon SVV infection (Figure 8C), particularly at 6 and 9 dpi and in proximity to inflammatory infiltrates.

**Figure 9:**
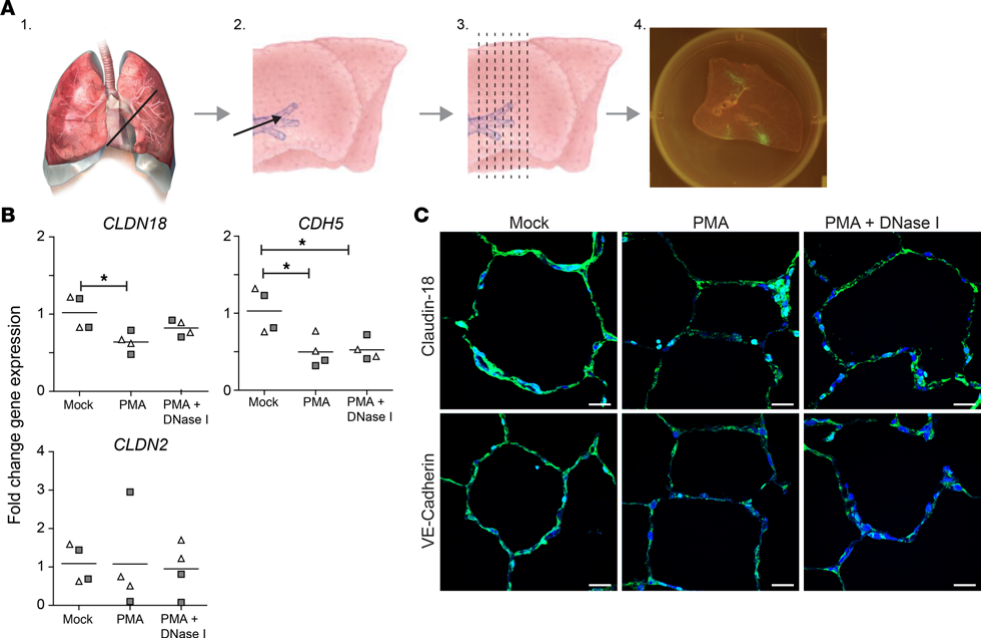
Neutrophil activation and NETs affect expression of alveolar epithelial and endothelial cell junction proteins in ex vivo macaque lung slice cultures. (**A**) Diagram showing generation of macaque lung slice cultures. Lungs were resected (step 1), inflated with agarose (step 2), cooled, and manually cut (step 3) to generate ± 1 mm–thick slices that can be cultured for > 7 days (step 4). Figure adapted and modified from ref. 71. (**B**) qPCR analysis of the indicated transcripts and *GAPDH* on rhesus macaque lung slices cultured with supernatant of autologous neutrophils treated with medium (mock), PMA, or both PMA and DNAse I. Lung slices were obtained from 2 animals (open triangles and gray squares), with 2 replicates analyzed per animal. Data are expressed as fold change in gene expression relative to control animals (calibrator) and normalized to *GAPDH* using the 2^–ΔΔCt^ method. Horizontal line indicates mean. *P < 0.05 by 1-way ANOVA and Bonferroni’s correction. (**C**) Lung slices were stained for claudin-18 or VE-cadherin (green). Nuclei were stained with Hoechst-33342 (blue). Scale bar: 20 μm.

Finally, we analyzed whether neutrophil activation and/or NETosis may contribute to impaired EEB integrity in macaque lung tissue. Ex vivo lung slice cultures (Figure 9A) from 2 rhesus macaques were exposed to supernatant obtained from autologous neutrophil cultures treated with medium, PMA to induce NET formation, or PMA and DNAse I to induce and subsequently degrade NETs. After 24 hours, *CLDN18*, *CDH5*, *CLDN2*, and *GAPDH* transcript levels were analyzed by qPCR, and claudin-18 and VE-cadherin protein expression was analyzed by IF staining. Claudin-18 transcript and protein levels were significantly reduced in lung slices incubated with supernatant from PMA-treated neutrophils (Figure 9, B and C), while DNase I treatment (partially) prevented claudin-18 downregulation. VE-cadherin transcript and protein levels were decreased in lung slices cultured with supernatant from PMA-treated or PMA + DNase I–cotreated neutrophils compared with mock-treated neutrophils (Figure 9, B and C). By contrast, none of the neutrophil supernatants affected *CLDN2* expression (Figure, B and C). These findings indicate that NETs decreased *CLDN18* expression, while other factors released by activated neutrophils caused downregulation of *CDH5* transcription. Overall, our results indicate that impaired alveolar EEB integrity is a pathogenic feature of varicella pneumonia in SVV-infected cynomolgus macaques and suggest the role of neutrophils and NETs in this process.

## Discussion

Varicella pneumonia is the most common and most severe complication of primary VZV infection in adults, and its pathogenesis is largely unknown. The current treatment regimen — antiviral therapy and, when necessary, supportive mechanical ventilation — still results in about 20% mortality and long-term impaired lung function (3, 42, 43). These data underscore the unmet need for novel intervention strategies that more effectively treat varicella pneumonia. In this study, we have used the well-established SVV nonhuman primate model that closely mimics human VZV disease (7, 8) to determine the mechanisms underlying the pathogenesis of varicella pneumonia. We performed detailed functional genomics combined with virological, histopathological, and immunological analyses on paired BAL and lung tissues of intratracheally SVV-infected cynomolgus macaques from 1 to 9 dpi. We report that SVV infection resulted in robust, transient virus replication that was controlled by innate antiviral immune responses in the infected lung. Additionally, the data demonstrate that the host inflammatory response involving neutrophil activation and NET formation, rather than the direct cytopathic effect of SVV, induced lung pathology that closely resembled varicella pneumonia in humans. We demonstrate that neutrophil activation directly affects expression of claudin-18 and VE-cadherin in nonhuman primate lung slice cultures, hereby providing a potential mechanism that may contribute to impaired alveolar EEB integrity observed in varicella-induced ALI.

SVV replication in the lung was rapidly controlled by innate antiviral immune responses before the anticipated impact of an adaptive immune response. SVV infection induced profound IFN-α/β responses, most likely produced by alveolar macrophages and plasmacytoid DCs (44–46). Although SVV actively evades antiviral activity of IFN-α/β in infected cells (47–49), secreted IFN-α/β induces ISGs in noninfected cells and stimulates the production of chemokines/cytokines by respiratory epithelial cells and lung-resident inflammatory cells (22, 50). Moreover, we demonstrate that the attracted and activated immune cells perpetuated local chemokine production, thereby suggesting a multifaceted pulmonary immune response in which the initial recruitment of neutrophils and NK cells — associated with the release of lytic granules, NET formation, and IFN-γ production — coincides with control of SVV replication. The subsequent influx of a high number of neutrophils, monocytes/macrophages, and T cells may further contribute to inhibition of SVV infection and clearance of virus-infected cells. However, robust SVV-specific T cell immunity does not appear until 10–14 dpi (46, 51), suggesting the limited role of T cells in controlling primary SVV infection in the lung.

We provide the first evidence to our knowledge that NETs are formed in response to a pulmonary herpesvirus infection. NETs are released by neutrophils undergoing NETosis, which can be induced by reactive oxygen species, binding of platelets to neutrophils, or recognition of viral danger-associated molecular patterns by pattern-recognition receptors expressed by neutrophils (52, 53). Virus-induced NET formation has been demonstrated for a broad range of respiratory viruses, mainly RNA viruses including influenza A virus, respiratory syncytial virus, and rhinovirus (35, 54, 55). Entrapment of viral particles or virus-infected cells by NETs restricts virus replication in vivo and in vitro (25, 52–55). Additionally, NETs may indirectly inhibit virus replication in vivo by activating immune cells (52, 53). Here, we showed that both SVV- and VZV-infected cells induce NET formation by human neutrophils, although the kinetics and abundance of NET formation in SVV-infected cynomolgus macaques suggest their limited role in repressing virus replication.

Varicella pneumonia–induced ALI is associated with alveolar lymphoplasmacytic inflammation, edema, fibrin deposition, necrosis, hemorrhaging, and loss of normal lung architecture of SVV-infected monkeys. Similar pathological changes are induced by other (respiratory) viruses, also often after cessation of virus replication (36, 56), suggesting a prominent role of local antiviral host responses rather than a direct SVV cytopathic effect on lung tissue. Indeed, we observed only small numbers of apoptotic epithelial cells during the peak of SVV infection. Impaired lung function results from extensive inflammation, compromised alveolar EEB integrity, and accumulation of edema and inflammatory cells in the alveolar lumen (24, 37). Here, we demonstrate that SVV infection was associated with downregulation of endothelial adherens junction molecule VE-cadherin and epithelial sealing TJ molecule claudin-18 at the RNA and protein level, while simultaneously inducing RNA expression of pore-forming claudin-2 and claudin-10, most likely contributing to the observed impaired EEB integrity. Multiple RNA viruses and a variety of inflammatory molecules and growth factors can downregulate VE-cadherin expression in endothelial cells (41, 57, 58), leading to alveolar edema formation and accumulation of inflammatory cells (40). Previous studies demonstrated an important role for IL-6–induced upregulation of claudin-2 and IL-1β/TNF-α/IFN-γ–induced downregulation of claudin-18 in increasing epithelial permeability (59, 60). Importantly, we demonstrate that neutrophil activation and NET formation were sufficient to decrease VE-cadherin and claudin-18 expression, respectively. Overall, the data indicate that inflammation — and especially neutrophils — contribute to ALI in varicella pneumonia.

Risk factors for developing life-threatening varicella pneumonia are incompletely understood. Smoking and pregnancy are associated with severe VZV pneumonia (61, 62). However, it remains challenging to predict the course of varicella pneumonia based on clinical signs at time of hospitalization (3, 43). Neutrophils and NETs play a central role in the pathogenesis of community acquired pneumonia (CAP) and its progression to ALI and ARDS (27, 63). High abundance of neutrophils and NETs in BAL samples of patients with both viral and nonviral CAP are associated with greater disease severity (27, 33, 36, 63, 64), and NET levels in paired plasma/serum samples are predictive for the clinical course of CAP (33). In line with these observations, we detected NETs in a subset of paired plasma and BAL samples during the course of self-limiting varicella pneumonia in SVV-infected animals. Consistent with prior studies, we observed NETs in BAL from influenza pneumonia patients (36) and — for the first time to our knowledge — demonstrate their presence in BAL of varicella pneumonia patients. We did not observe a consistent correlation between NET levels in paired BAL and plasma samples, nor between NET abundance and disease outcome, in the 3 VZV pneumonia patients analyzed. However, given that our study was limited by the inclusion of a small number of severe varicella pneumonia patients, the potential use of NET abundance in BAL samples as a diagnostic marker for disease severity and prognosis needs to be further evaluated in a larger cohort of patients with VZV pneumonia.

In conclusion, we provide detailed insight into the virus and host factors involved in the acute phase of varicella pneumonia. While antiviral therapy is critical to reduce varicella pneumonia mortality (65), our study suggests that the prognosis of varicella pneumonia may be improved by simultaneously targeting pulmonary immune responses. The benefit of nonspecific immune suppression (corticosteroids) in varicella pneumonia patients remains controversial (6, 66). However, specific targeting of potential pathogenic innate immune cells involved in the pathogenesis of ALI/ARDS, especially neutrophils, provides an exciting avenue to explore to reduce morbidity and mortality caused by varicella pneumonia. Notably, therapeutic inhibition of NET production and inactivation of NET components — such as recombinant human DNase I (Pulmozyme) used to treat cystic fibrosis patients (67) — are currently explored to improve the outcome of pneumonia caused by other pathogens (26, 55, 68).

## Methods

### Cells and viruses.

Low-passage WT SVV (Delta herpesvirus strain) and recombinant SVV-EGFP–expressing EGFP fused to the N-terminus of ORF66 were cultured on BS-C-1 cells as described (18, 47) (Supplemental Methods). VZV-EGFP ectopically expresses EGFP and was cultured on ARPE-19 cells (47, 69).

### Animal experiments.

Twenty juvenile (2 years old) male SVV-seronegative cynomolgus macaques (*Macaca fascicularis*), carrying i.p. implanted temperature transponders (Star-Oddi Ltd.), were intratracheally inoculated with 8.5 × 10^5^ plaque forming units (PFU) of SVV (*n* = 15) or an equivalent number of uninfected BS-C-1 cells diluted in 5 mL of PBS (*n* = 15) (Supplemental Methods). SVV-infected animals were euthanized at 3 dpi (*n* = 5; group 1), 6 dpi (*n* = 5; group 2), and 9 dpi (*n* = 5; group 3). Mock-infected animals were euthanized at 3 dpi (*n* = 5; group 4). Blood samples were collected at 0, 1, 2, and 3 dpi (groups 1 and 4); at 0, 2, 4, and 6 dpi (group 2); and at 0, 3, 5, 7, 8, and 9 dpi (group 3). BAL was performed at 1, 2, and 3 dpi (group 1); at 2, 4, and 6 dpi (group 2); at 5, 7, and 9 dpi (group 3); and at 3 dpi (group 4). Tissues collected at necropsy were formalin fixed and paraffin embedded (FFPE), snap-frozen in liquid nitrogen, or preserved in RNAlater solution (Thermo Fisher Scientific) and stored at –80°C.

### Virus isolation and titration.

BAL cells were incubated for 30 minutes at 37°C in 1 mL RPMI 1640 (Thermo Fisher Scientific) containing 10% FBS (R10F) and 1 μg/mL Phytohemagglutinin-L (MilliporeSigma), after which 10-fold serial dilutions of BAL cells in R10F were added to semiconfluent BS-C-1 cells in 96-well plates (*n* = 8 replicates). After 7 days, infectious virus titers were calculated using the Spearman-Karber method and converted to PFU (PFU = 0.69 × TCID_50_).

### Nucleic acid isolation and qPCR.

DNA was extracted from PBMC, BAL samples, and frozen lung tissue using the QIAamp DNA Mini Kit (QIAGEN). RNA extraction was performed by homogenizing pooled frozen lung biopsies (*n* = 3 per animal) in 1 mL TRIzol reagent (Invitrogen) using the MP Fastprep-24 (MP Biomedicals) and the RNeasy Mini Kit (QIAGEN). cDNA synthesis was performed using 5 μg total RNA and Superscript IV reverse transcriptase (Invitrogen) with oligo(dT) primers (Invitrogen). qPCR was performed in duplicate on an ABI Prism 7500 using TaqMan 2× Universal Master Mix (Applied Biosystems). DNA qPCR was performed with primers and probes specific for SVV *ORF21* and the single copy gene *OSM* (70) (Supplemental Table 2). cDNA qPCR was performed with primers and probes specific for SVV *ORF63* (17) (Supplemental Table 2), *OSM* (70), *CXCL10* (Mf02788358_g1) (47), *CDH5* (Mf00901470_m1), *CLDN2* (Mf04369164_m1), *CLDN10* (MF02862371_m1), *CLDN18* (Mf02805373_m1), *DDX58* (Mf02789247_m1), *GAPDH* (Mf04392546_g1), *IFIT1* (Mf04355804_m1), *IFN-α2* (Mf04256335_s1), *IFN-γ* (Mf02788577_m1), *MX1* (Mf00895608_m1), and *STAT1* (Hs01013996_m1) (Applied Biosystems).

### RNA labeling and microarray analysis.

Total RNA (200 ng) was labeled using the MessageAmp Premier RNA Amplification kit (Applied Biosystems) and hybridized to Rhesus Macaque genome–specific GeneChips (Affymetrix), according to the manufacturer’s recommendations. Image analysis was performed using GeneChip Operating Software (Affymetrix). Microarray data analysis is described in Supplemental Methods. Raw data have been deposited in the ArrayExpress database under accession number E-MTAB-8485 (https://www.ebi.ac.uk/arrayexpress/experiments/E-MTAB-8485/).

### Flow cytometry.

BAL cells were stained using fluorochrome-conjugated mouse monoclonal antibodies directed to human CD11b-PE (clone D12), CD45-PerCP (TU166), CD20–PE-Cy7 (L27), CD16-AF647 (3G3), CD3–APC-Cy7 (SP34-2),CD8-AmCyan (SK1), CD95-PE (DX-2), CD69-PerCP (FN50), CCR4–PE-Cy7 (1G1), CD28-APC (CD28.2), and CD4-V450 (L200) (All from BD Biosciences), as well as HLA-DR–Pacific Blue (L243; BioLegend). Fluorescence was measured on a FACS Canto II and analyzed using BD FACS Diva software (both BD Biosciences) and FlowJo software (Tree Star Inc.) (Supplemental Methods, Supplemental Figure 4). Extrapolated cell number was determined for each of the analyzed cell types present in BAL fluid as follows: “number of BAL cells/mL” × “frequency of the population.”

### In situ analyses.

FFPE tissue sections were analyzed by H&E staining, IHC, immunofluorescence (IF), and ISH. IHC and IF was performed using the following primary antibodies: mouse anti-CD3 (clone F7.2.38), anti-CD20 (L26), anti-CD68 (KP1), anti-granzyme B (GrB-7), anti–neutrophil elastase (NP57), anti–CD45 (2B11 + PD7/26), and anti-CD31 (JC70A) (all from Dako); anti-cytokeratin (AE1/AE3) and anti-pSTAT1 (Tyr701; clone ST1P-11A5) (Thermo Fisher Scientific); goat anti-MPO (AF3667; R&D systems); rabbit anti-citH3 (ab5103), anti–claudin-18 (34H14L15), and anti–VE cadherin (ab33168) (Abcam); and anti–IFN-α (PBL Assay Science) as described in Supplemental Methods. ISH was performed using probes directed to SVV *ORF63* (71), *CCL2*, *CXCL8*, *CXCL10*, ubiquitin C (positive control), and the bacterial gene *dapB* (negative control) using the RNAScope 2.5 HD Assay (Advanced Cell Diagnostics) (Supplemental Methods).

### Pathology scoring.

H&E-stained slides of trachea, primary bronchus, and lung (*n* ≥ 2 lung samples per animal) were examined by experienced pathologists in a blinded fashion and scored the overall level of inflammation, necrosis, hemorrhage, fibrosis, emphysema, and the presence of type II pneumocytes, on a scale from 0 to 3 (0, none; 1, mild; 2, moderate; 3, severe) (Supplemental Methods).

### Neutrophils and quantification of NET formation.

Neutrophils were isolated from human and rhesus macaque blood by density gradient centrifugation using Polymorphprep (Axis-Shield). Neutrophils were cultured in RPMI 1640 supplemented with 2% FBS and antibiotics (R2F medium). To quantify in vitro NET formation, glass coverslips in 24-well plate wells were coated with poly-D lysine (50 μg/mL) for 2 hours at 37°C, washed, and air-dried. Neutrophils were plated at 2 × 10^5^ cells/well in 500 μL R2F, treated with PMA (500 nM; MilliporeSigma), or cocultured with either mock- and SVV-EGFP–infected BS-C-1 cells or mock- and VZV-EGFP–infected ARPE-19 cells (2 × 10^5^ cells/well) for 6 hours at 37°C. Neutrophils were fixed with 4% PFA dissolved in PBS, permeabilized using 0.1% Triton-X100 dissolved in PBS, and stained for DNA (Hoechst) and neutrophil elastase (clone NP57). Experiments were performed on 2 human donors, and 3 biological replicates were analyzed. NETs were quantified by confocal microscopy.

### Ex vivo lung slice cultures.

Lungs from 2 rhesus macaques (*Macaca mulatta*; female, ages 10 and 16 years old), obtained from the Biomedical Primate Research Centre (Rijswijk, The Netherlands) were inflated with 4% low-melting 2-hydroxyethyl agarose (MilliporeSigma) dissolved in a 1:1 ratio of PBS and DMEM supplemented with 10% heat-inactivated FBS (MilliporeSigma) and antibiotics (Lonza). Lung slices of approximately 1 mm thick were manually cut (Figure 9A) and cultured in 6-well plate wells as described (72, 73). Neutrophils (8 × 10^6^ in 8 mL R2F/well of a 6-well plate) were incubated with medium or PMA (500 nM; MilliporeSigma) for 6 hours. Some samples were additionally incubated with DNase I (2,000 units/mL; MilliporeSigma) for 30 minutes. Supernatant of neutrophils treated with medium, PMA, or PMA and DNase was added to the ex vivo lung slices (1 mL supernatant added to 4 mL medium per well), and slices were cultured for an additional 24 hours. Samples were stored in TRIzol for subsequent RNA extraction and qPCR analysis.

### Detection of His-DNA complexes.

Clarified BAL supernatant (500*g*, 10 minutes) and plasma/serum samples were analyzed using the Human Cell Death Detection ELISA^PLUS^ (MilliporeSigma). Cynomolgus macaque samples were analyzed using ABTS as a substrate and measured at 405 nm and 492 nm as a reference. Human samples were analyzed using 3,3’,5,5’-Tetramethylbenzidine (TMB) as a substrate and measured at 450 nm, using 620 nm as a reference. For each species, all samples were analyzed in the same experiment and on the same plate.

### Detection of MPO-DNA complexes.

BAL and plasma/serum samples were analyzed for the presence of MPO-DNA complexes as described (35). In brief, wells were coated with mouse anti-MPO antibody (5 μg/mL; clone 4A4, Bio-Rad) overnight at 4°C. Samples (20 μL) were mixed with incubation buffer and peroxidase-labeled anti-DNA antibody (80 μL/sample; both from the Human Cell Death Detection ELISA^PLUS^ kit; MilliporeSigma), incubated for 2 hours at room temperature, and analyzed using TMB as a substrate. All analyzed macaque and human samples were measured on the same plate and in the same assay. Pooled plasma from healthy human blood donors and supernatant of PMA-treated human neutrophils were included as negative and positive control, respectively.

### Statistics.

GraphPad Prism 5.0 (GraphPad Software Inc.) was used for statistical analyses. Data were analyzed using 1-way ANOVA corrected for multiple comparisons by Bonferroni’s adjustment (Figure 2, B–D; Figure 3B; Figure 4, B–D; Figure 6F; Figure 8B; Figure 9C; and Supplemental Figure 1, A and B) or unpaired 2-tailed Student’s *t* test (Figure 6D). *P* < 0.05 were considered significant.

### Study approval.

Surplus BAL, plasma, and serum samples from VZV and influenza pneumonia patients obtained for diagnostic purposes at the Erasmus MC (Rotterdam, the Netherlands) were used. Data collection and analyses were conducted on anonymized samples, which was approved by the medical ethical committee of the Erasmus MC (MEC-2015-306). Informed consent was waived by the institutional privacy knowledge officer. Animal experiments were performed in strict compliance with European guidelines (EU Directive on Animal Testing 2010/63/EU) and Dutch legislation (Experiments on Animals Act, 1997). The study protocol was approved by the independent animal experimentation ethical review committee *Stichting Dier Experimenten Commissie Consult* (DEC Consult, permit numbers EMC2975 [cynomolgus macaques] and BPRC permit number 7962 [rhesus macaques]). The manuscript text was written in accordance with the Animal Research: reporting of In Vivo Experiments (ARRIVE) guidelines.

## Author contributions

WJDO and GMGMV conceived and designed the study. WJDO, GPVN, TM, FZB, and RDDV conducted the experiments. WJDO, HJVDH, MWD, JJAVK, WFJVI, JMAVDB, and ACA analyzed the data. WJDO and GMGMV wrote the manuscript.

## Supplementary Material

Supplemental data

## Version 1 10/06/2020

In-Press Preview

## Version 2 11/05/2020

Electronic publication

## Version 3 03/18/2021

Figure changes

## References

[1] Virgin HW, Wherry EJ, Ahmed R (2009). Redefining chronic viral infection. Cell.

[2] Gershon AA (2015). Varicella zoster virus infection. Nat Rev Dis Primers.

[3] Mohsen AH, McKendrick M (2003). Varicella pneumonia in adults. Eur Respir J.

[4] Navaratnam AMD, Ma N, Farrukh M, Abdulla A (2017). Chickenpox: an ageless disease. BMJ Case Rep.

[5] WEBER DM, PELLECCHIA JA (1965). VARICELLA PNEUMONIA: STUDY OF PREVALENCE IN ADULT MEN. JAMA.

[6] Mirouse A (2017). Severe varicella-zoster virus pneumonia: a multicenter cohort study. Crit Care.

[7] Haberthur K, Messaoudi I (2013). Animal models of varicella zoster virus infection. Pathogens.

[8] Ouwendijk WJ, Verjans GM (2015). Pathogenesis of varicelloviruses in primates. J Pathol.

[9] Sawyer MH, Chamberlin CJ, Wu YN, Aintablian N, Wallace MR (1994). Detection of varicella-zoster virus DNA in air samples from hospital rooms. J Infect Dis.

[10] Zerboni L, Sen N, Oliver SL, Arvin AM (2014). Molecular mechanisms of varicella zoster virus pathogenesis. Nat Rev Microbiol.

[11] Feldman S (1994). Varicella-zoster virus pneumonitis. Chest.

[12] Ranney EK, Norman MG, Silver MD (1967). Varicella pneumonitis. Can Med Assoc J.

[13] Dueland AN (1992). Acute simian varicella infection. Clinical, laboratory, pathologic, and virologic features. Lab Invest.

[14] Gray WL, Williams RJ, Chang R, Soike KF (1998). Experimental simian varicella virus infection of St. Kitts vervet monkeys. J Med Primatol.

[15] Gray WL, Starnes B, White MW, Mahalingam R (2001). The DNA sequence of the simian varicella virus genome. Virology.

[16] Mahalingam R (2007). Simian varicella virus reactivation in cynomolgus monkeys. Virology.

[17] Messaoudi I (2009). Simian varicella virus infection of rhesus macaques recapitulates essential features of varicella zoster virus infection in humans. PLoS Pathog.

[18] Ouwendijk WJ (2013). T-Cell tropism of simian varicella virus during primary infection. PLoS Pathog.

[19] Meyer C, Kerns A, Barron A, Kreklywich C, Streblow DN, Messaoudi I (2011). Simian varicella virus gene expression during acute and latent infection of rhesus macaques. J Neurovirol.

[20] Mi H (2017). PANTHER version 11: expanded annotation data from Gene Ontology and Reactome pathways, and data analysis tool enhancements. Nucleic Acids Res.

[21] Mi H (2019). Protocol Update for large-scale genome and gene function analysis with the PANTHER classification system (v.14.0). Nat Protoc.

[22] McNab F, Mayer-Barber K, Sher A, Wack A, O’Garra A (2015). Type I interferons in infectious disease. Nat Rev Immunol.

[23] Matthay MA, Ware LB, Zimmerman GA (2012). The acute respiratory distress syndrome. J Clin Invest.

[24] Matthay MA (2019). Acute respiratory distress syndrome. Nat Rev Dis Primers.

[25] Ashar HK (2018). The Role of Extracellular Histones in Influenza Virus Pathogenesis. Am J Pathol.

[26] Lefrançais E, Mallavia B, Zhuo H, Calfee CS, Looney MR (2018). Maladaptive role of neutrophil extracellular traps in pathogen-induced lung injury. JCI Insight.

[27] Potey PM, Rossi AG, Lucas CD, Dorward DA (2019). Neutrophils in the initiation and resolution of acute pulmonary inflammation: understanding biological function and therapeutic potential. J Pathol.

[28] Moorthy AN (2016). Capsules of virulent pneumococcal serotypes enhance formation of neutrophil extracellular traps during in vivo pathogenesis of pneumonia. Oncotarget.

[29] Brinkmann V (2004). Neutrophil extracellular traps kill bacteria. Science.

[30] Remijsen Q, Kuijpers TW, Wirawan E, Lippens S, Vandenabeele P, Vanden Berghe T (2011). Dying for a cause: NETosis, mechanisms behind an antimicrobial cell death modality. Cell Death Differ.

[31] Brinkmann V, Abu Abed U, Goosmann C, Zychlinsky A (2016). Immunodetection of NETs in Paraffin-Embedded Tissue. Front Immunol.

[32] Wang Y (2009). Histone hypercitrullination mediates chromatin decondensation and neutrophil extracellular trap formation. J Cell Biol.

[33] Ebrahimi F (2018). Markers of neutrophil extracellular traps predict adverse outcome in community-acquired pneumonia: secondary analysis of a randomised controlled trial. Eur Respir J.

[34] Thomas GM (2012). Extracellular DNA traps are associated with the pathogenesis of TRALI in humans and mice. Blood.

[35] Kessenbrock K (2009). Netting neutrophils in autoimmune small-vessel vasculitis. Nat Med.

[36] Zhu L (2018). High Level of Neutrophil Extracellular Traps Correlates With Poor Prognosis of Severe Influenza A Infection. J Infect Dis.

[37] Short KR, Kroeze EJBV, Fouchier RAM, Kuiken T (2014). Pathogenesis of influenza-induced acute respiratory distress syndrome. Lancet Infect Dis.

[38] Khan N, Asif AR (2015). Transcriptional regulators of claudins in epithelial tight junctions. Mediators Inflamm.

[39] Koval M (2013). Claudin heterogeneity and control of lung tight junctions. Annu Rev Physiol.

[40] Corada M (1999). Vascular endothelial-cadherin is an important determinant of microvascular integrity in vivo. Proc Natl Acad Sci U S A.

[41] Vestweber D, Winderlich M, Cagna G, Nottebaum AF (2009). Cell adhesion dynamics at endothelial junctions: VE-cadherin as a major player. Trends Cell Biol.

[42] BOCLES JS, EHRENKRANZ NJ, MARKS A (1964). ABNORMALITIES OF RESPIRATORY FUNCTION IN VARICELLA PNEUMONIA. Ann Intern Med.

[43] Mohsen AH, Peck RJ, Mason Z, Mattock L, McKendrick MW (2001). Lung function tests and risk factors for pneumonia in adults with chickenpox. Thorax.

[44] (2015). Alveolar macrophage-derived type I interferons orchestrate innate immunity to RSV through recruitment of antiviral monocytes. J Exp Med.

[45] Arnold N, Girke T, Sureshchandra S, Nguyen C, Rais M, Messaoudi I (2016). Genomic and functional analysis of the host response to acute simian varicella infection in the lung. Sci Rep.

[46] Haberthur K, Meyer C, Arnold N, Engelmann F, Jeske DR, Messaoudi I (2014). Intrabronchial infection of rhesus macaques with simian varicella virus results in a robust immune response in the lungs. J Virol.

[47] Ouwendijk WJD, van Veen S, Mahalingam R, Verjans GMGM (2017). Simian varicella virus inhibits the interferon gamma signalling pathway. J Gen Virol.

[48] Verweij MC (2015). Varicella Viruses Inhibit Interferon-Stimulated JAK-STAT Signaling through Multiple Mechanisms. PLoS Pathog.

[49] Whitmer T, Malouli D, Uebelhoer LS, DeFilippis VR, Früh K, Verweij MC (2015). The ORF61 Protein Encoded by Simian Varicella Virus and Varicella-Zoster Virus Inhibits NF-κB Signaling by Interfering with IκBα Degradation. J Virol.

[50] Platanias LC (2005). Mechanisms of type-I- and type-II-interferon-mediated signalling. Nat Rev Immunol.

[51] Haberthur K (2013). Genome-wide analysis of T cell responses during acute and latent simian varicella virus infections in rhesus macaques. J Virol.

[52] Boeltz S (2019). To NET or not to NET:current opinions and state of the science regarding the formation of neutrophil extracellular traps. Cell Death Differ.

[53] Schönrich G, Raftery MJ (2016). Neutrophil Extracellular Traps Go Viral. Front Immunol.

[54] Cortjens B (2016). Neutrophil extracellular traps cause airway obstruction during respiratory syncytial virus disease. J Pathol.

[55] Toussaint M (2017). Host DNA released by NETosis promotes rhinovirus-induced type-2 allergic asthma exacerbation. Nat Med.

[56] Gralinski LE, Baric RS (2015). Molecular pathology of emerging coronavirus infections. J Pathol.

[57] Shrivastava-Ranjan P, Rollin PE, Spiropoulou CF (2010). Andes virus disrupts the endothelial cell barrier by induction of vascular endothelial growth factor and downregulation of VE-cadherin. J Virol.

[58] Singh S, Anupriya MG, Modak A, Sreekumar E (2018). Dengue virus or NS1 protein induces trans-endothelial cell permeability associated with VE-Cadherin and RhoA phosphorylation in HMEC-1 cells preventable by Angiopoietin-1. J Gen Virol.

[59] Fang X, Neyrinck AP, Matthay MA, Lee JW (2010). Allogeneic human mesenchymal stem cells restore epithelial protein permeability in cultured human alveolar type II cells by secretion of angiopoietin-1. J Biol Chem.

[60] Suzuki T, Yoshinaga N, Tanabe S (2011). Interleukin-6 (IL-6) regulates claudin-2 expression and tight junction permeability in intestinal epithelium. J Biol Chem.

[61] Grayson ML, Newton-John H (1988). Smoking and varicella pneumonia. J Infect.

[62] Harger JH (2002). Risk factors and outcome of varicella-zoster virus pneumonia in pregnant women. J Infect Dis.

[63] Cheng OZ, Palaniyar NET balancing: a problem in inflammatory lung diseases. Front Immunol.

[64] Grommes J, Soehnlein O (2011). Contribution of neutrophils to acute lung injury. Mol Med.

[65] Haake DA, Zakowski PC, Haake DL, Bryson YJ (1990). Early treatment with acyclovir for varicella pneumonia in otherwise healthy adults: retrospective controlled study and review. Rev Infect Dis.

[66] Mer M, Richards GA (1998). Corticosteroids in life-threatening varicella pneumonia. Chest.

[67] Fuchs HJ (1994). Effect of aerosolized recombinant human DNase on exacerbations of respiratory symptoms and on pulmonary function in patients with cystic fibrosis. The Pulmozyme Study Group. N Engl J Med.

[68] Kolaczkowska E (2015). Molecular mechanisms of NET formation and degradation revealed by intravital imaging in the liver vasculature. Nat Commun.

[69] Zhang Z (2010). Genome-wide mutagenesis reveals that ORF7 is a novel VZV skin-tropic factor. PLoS Pathog.

[70] Ouwendijk WJ (2012). Simian varicella virus infection of Chinese rhesus macaques produces ganglionic infection in the absence of rash. J Neurovirol.

[71] Ouwendijk WJD, van Veen S, Mehraban T, Mahalingam R, Verjans GMGM (2018). Simian Varicella Virus Infects Enteric Neurons and α4β7 Integrin-Expressing Gut-Tropic T-Cells in Nonhuman Primates. Viruses.

[72] De Vries RD, Rennick LJ, Duprex WP, De Swart RL (2018). Paramyxovirus Infections in Ex Vivo Lung Slice Cultures of Different Host Species. Methods Protoc.

[73] Nguyen DT (2013). Paramyxovirus infections in ex vivo lung slice cultures of different host species. J Virol Methods.

