# Supplementary material for: Alveolar barrier disruption in varicella pneumonia is associated with neutrophil extracellular trap formation
Source: JCI Insight. 2020 Nov 5;5(21):e138900. doi: 10.1172/jci.insight.138900 (PMC7710321; doi:10.1172/jci.insight.138900)
Supplement: Supplemental data [file jciinsight-5-138900-s010.pdf]

SUPPLEMENTAL INFORMATION

Supplemental Materials and Methods

Animal experiments. Animals were housed in groups, received standard primate feed and fresh fruit on a daily basis, and had access to water *ad libitum*. Cages also contained multiple sources of environmental enrichment. During the experiments, animals were housed in HEPA-filtered negatively pressurized ABSL-3 isolator cages. Animal welfare was checked on a daily basis, and all animal handling was performed under anesthesia (ketamine and medetomidine) to minimize animal discomfort. After handling, atipamezole was administered to antagonize the effect of medetomidine. Prior to inoculation the abdomen, thorax and back of the animals were shaved to allow careful examination for skin rash on sample days. Blood samples were collected in VACUETTE tubes (Greiner Bio-One) containing K_3EDTA as an anticoagulant. BAL was performed by intratracheal administration and collection of 10 ml PBS through a flexible catheter. At necropsy, BAL samples were obtained by direct administration of 10 ml PBS in the right-hand side of the lung. BAL samples were centrifuged for 10 min at 350 x g, supernatant was stored at -20°C and cells were dissolved in RPMI-1640 medium (Lonza) supplemented with 10% FBS and antibiotics (R10F medium). When necessary, BAL cells were incubated with Red Blood Cell Lysis Buffer (Roche) prior to addition of R10F. Cells were used for virus isolation, DNA isolation and flow cytometry.

Cells and viruses. African green monkey kidney epithelial BS-C-1 cells and human retinal pigment epithelial ARPE-19 cells were obtained from the American Type Culture Collection (ATCC catalogue numbers CCL-26 and CRL-2302, respectively). BS-C-1 cells were cultured in Dulbecco's Modified Eagle Medium (DMEM) (Lonza) supplemented with 10% heat-inactivated

fetal bovine serum (FBS; Sigma) and antibiotics (Lonza) at 37ºC in a CO_2 incubator. ARPE-19 cells were cultured in a 1:1 ratio (vol/vol) of DMEM and Ham's F12 nutrient mixture (both Lonza) supplemented with 10% FBS and antibiotics at 37ºC in a CO_2 incubator. A low-passage clinical isolate of the Delta herpesvirus strain of SVV, isolated from PBMC of an acutely infected African green monkey (AGM269 in (1)) and propagated 5 times in BS-C-1 cells to generate virus stocks used for inoculation of cynomolgus macaques. Equivalent numbers of uninfected BS-C-1 cells, of the same passage number as used for virus stock preparation, were cryopreserved to be used for mock infection. Virus stocks were titrated in triplicate by co-cultivation of 10-fold serial dilutions of thawed SVV-infected BS-C-1 cells on uninfected monolayers of BS-C-1 cells grown in 6 well plates. Plates were fixed at 72 hours post-infection (hpi) for 15 minutes in 10% neutral buffered formalin and plaques were visualized by incubating cells for 5 minutes with a 2% crystal violet solution. Recombinant SVV-EGFP expresses enhanced green fluorescent protein (EGFP) fused to the N-terminus of ORF66, VZV-EGFP ectopically expresses EGFP and both viruses are not attenuated in cell culture. VZV-EGFP was cultured on ARPE-19 cells as described (2, 3).

Analysis of microarray data. Probe-level data was normalized using quantile normalization and the transformed probe values were summarized into probe set values by the median polish method (4). Probe set wise comparisons between the experimental conditions were performed using Limma (5). Correction for multiple testing was achieved by applying a false discovery rate (FDR) of 0.05, calculated using the Benjamini–Hochberg procedure. Values in heatmaps represent $\log_2$-fold change in gene expression for individual animals relative to the average expression level of that specific gene in the control group. Data processing and principal component was performed in R, a language for statistical computation (6). Gene set enrichment analysis was performed using the Gene Ontology PANTHER enrichment test and rhesus macaque (*Macaca mulatta*) reference list to identify statistically significant (FDR < 0.05) Gene Ontology biological processes (complete) (7, 8). Additional pathway analyses were performed

using DAVID Bioinformatics Resources version 6.8 (9, 10), Reactome Pathway Knowledgebase (11) and Ingenuity Pathway Analysis (Qiagen) to identify gene categories of interest. Heatmaps were generated using TreeView 3.0, using the uncentered Pearson correlation as a distance metric with average linkage for the hierarchical clustering (12).

Flow cytometry gating strategy. Viable single cells were selected based on forward scatter area (FSC-A) and FSC-height signal intensity followed by FSC-A and sideward scatter area and gated on $CD45^{pos}$ leukocytes and $CD45^{neg}$ non-leukocytes. Large $CD45^{pos}$ leukocytes were defined as monocytes/macrophages ($CD11b^{pos}$ $HLA\text{-}DR^{high}$) and granulocytes ($CD11b^{pos}$ $HLA\text{-}DR^{dim}$) (13). Small $CD45^{pos}$ leukocytes were identified as B-cells ($CD20^{pos}$ $HLA\text{-}DR^{pos}$), NK cells ($CD3^{neg}$ $CD8^{pos}$ $CD16^{pos/neg}$), DCs ($CD3^{neg}$ $CD16^{neg}$ $CD20^{neg}$ $HLA\text{-}DR^{pos}$ $CD11b^{pos/neg}$) and T-cells ($CD3^{pos}$) (Fig. S4A). T-cells were further categorized into CD4 ($CD3^{pos}$ $CD4^{pos}$) and CD8 ($CD3^{pos}$ $CD8^{pos}$) T-cells and the respective T-cell subsets naïve ($CD28^{pos}$ $CD95^{neg}$), central memory ($CD28^{pos}$ $CD95^{pos}$) and effector memory ($CD28^{neg}$ $CD95^{pos}$) T-cells (14) (Fig. S4B).

In situ analyses. FFPE tissue sections (4µm thick) were deparaffinized, rehydrated, subjected to heat-induced antigen retrieval in either citrate buffer (10 mM, p=6.0) or Tris-EDTA buffer (10 mM, pH=9.0), blocked and incubated with primary antibodies overnight at 4°C, as described (1). IHC staining was performed by incubating sections with biotin-conjugated secondary goat anti-mouse or goat anti-rabbit IgG antibodies followed by horseradish peroxidase-conjugated streptavidin (Dako). Staining was visualized using 3-amino-9-ethylcarbazole (AEC), and sections were counterstained with hematoxylin (both: Sigma-Aldrich). Immunofluorescent staining was performed using secondary Alexa Fluor 488 (AF488)-conjugated goat anti-rabbit Ig, AF488-conjugated chicken anti-rabbit Ig, AF555-conjugated goat anti-mouse Ig, AF594-conjugated goat anti-mouse Ig and AF594-conjugated rabbit anti-goat Ig antibodies. Nuclei were stained with Hoechst-33342 (Sigma Aldrich) and sections were mounted in Prolong Diamond Antifade

Reagent (Thermo Fisher Scientific). ISH staining was visualized using FastRed as a substrate, and slides were counterstained with hematoxylin and mounted with Ecomount (Biocare Medical). The ApopTag® Fluorescein In Situ Apoptosis Detection Kit (Millipore) was used to detect apoptotic cells in tissue sections, as per manufacturer's instructions. Subsequently sections were treated with ddH2O containing 3% H2O2 and 1% methanol for 30 minutes at room temperature prior to immunofluorescent staining as described above. Fluorescent stainings were analyzed on a Zeiss LSM700 confocal laser scanning microscope (Zeiss) and using ZEN 2010 software (Zeiss) to adjust brightness and contrast. Images of IHC and ISH pictures were obtained by scanning the slide using the Nanozoomer 2.0 HT (Hamamatsu) or Olympus ColorView camera fitted onto an Olympus BX51 microscope using Olympus Cell A software.

Pathology scoring. Each tissue was given a score for the following pathological changes: overall level of inflammation (determined by the amount of inflammatory cells present and the amount of tissue involved), necrosis (characterized by an eosinophilic background containing karyorrhectic and karyolitic debris associated with high numbers of infiltrating neutrophils), hemorrhage, fibrosis, emphysema and the presence of type II pneumocytes - on a scale from 0 – 3 (0 = none; 1 = mild; 2 = moderate; 3 = severe). Where inflammation was present the dominant cell type was noted. An overall description of lesions found was also recorded.

Supplemental References

1. Ouwendijk WJD *et al.* T-Cell Tropism of Simian Varicella Virus during Primary Infection. *PLoS Pathog.* 2013;9:e1003368.
2. Ouwendijk WJD, Van Veen S, Mahalingam R, Verjans GMGM. Simian varicella virus inhibits the interferon gamma signalling pathway. *J. Gen. Virol.* 2017;98:2582-2588.
3. Zhang Z *et al.* Genome-wide mutagenesis reveals that ORF7 is a novel VZV skin-tropic factor. *PLoS Pathog.* 2010;6:e1000971.

4. Gautier L, Cope L, Bolstad BM, Irizarry RA, affy--analysis of Affymetrix GeneChip data at the probe level. *Bioinformatics* 2004;20:307-315.

5. Ritchie ME *et al.* limma powers differential expression analyses for RNA-sequencing and microarray studies. *Nucleic Acids Res.* 2015;43:e47.

6. R. Core Team (2018) R: A Language and Environment for Statistical Computing. (https://www.R-project.org).

7. Mi H *et al.* PANTHER version 11: expanded annotation data from Gene Ontology and Reactome pathways, and data analysis tool enhancements. *Nucleic Acids Res.* 2017;45:D183-D189.

8. Mi H *et al.* Protocol Update for large-scale genome and gene function analysis with the PANTHER classification system (v.14.0). *Nat. Protoc.* 2019;14:703-721.

9. Huang DW, Sherman BT, Lempicki RA. Systematic and integrative analysis of large gene lists using DAVID bioinformatics resources. *Nat. Protoc.* 2009;4:44-57.

10. Huang DW, Sherman BT, Lempicki RA. Bioinformatics enrichment tools: paths toward the comprehensive functional analysis of large gene lists. *Nucleic Acids Res.* 2009;37:1-13.

11. Fabregat a *et al.* The Reactome Pathway Knowledgebase. *Nucleic Acids Res.* 2018;46: D649-D655.

12. Keil C *et al.* (2016) Treeview 3.0 (beta 1) - Visualization and analysis of large data matrices. (https://bitbucket.org/TreeView3Dev/treeview3/).

13. Cai Y *et al.* In vivo characterization of alveolar and interstitial lung macrophages in rhesus macaques: implications for understanding lung disease in humans. *J. Immunol.* 2014;192:2821-2829.

14. Pitcher CJ *et al.* Development and Homeostasis of T Cell Memory in Rhesus Macaque. *The J. Immunol.* 2002;168:29-43.

Supplemental Figures, Figure Legends and Tables

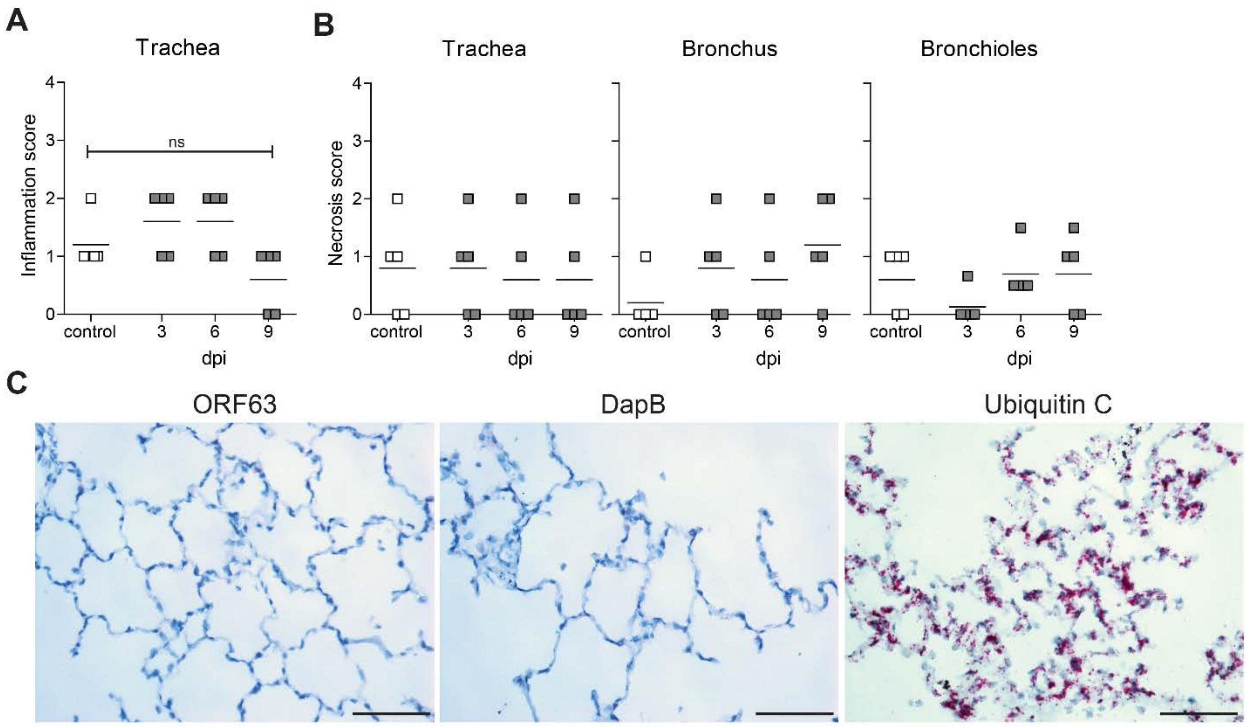

Supplemental Figure 1. Detection of histopathological changes and SVV ORF63 RNA in lung tissue of SVV-infected cynomolgus macaques. *(***A, B**) Tissue sections were examined by staining with hematoxylin and eosin (H&E) and scored for inflammation (**A**) and necrosis (**B**). ns, not significant by one-way ANOVA and Bonferroni correction. A mild inflammatory response was observed in the tracheal submucosa of both mock- and SVV-infected animals, most likely due to the intratracheal inoculation procedure. (**C**) Specificity of SVV ORF63 RNA ISH. Lung sections from mock-infected cynomolgus macaques were stained for SVV ORF63 RNA, bacterial dihydrodipicolinate reductase transcript (DapB, negative control) and ubiquitin C RNA by ISH (red/pink signal). Nuclei were stained with hematoxylin (blue). No SVV ORF63 or DapB ISH signal was detected in lung sections from control animals, whereas the universally expressed ubiquitin C was always readily detectable. Scale bar: 100 µm.

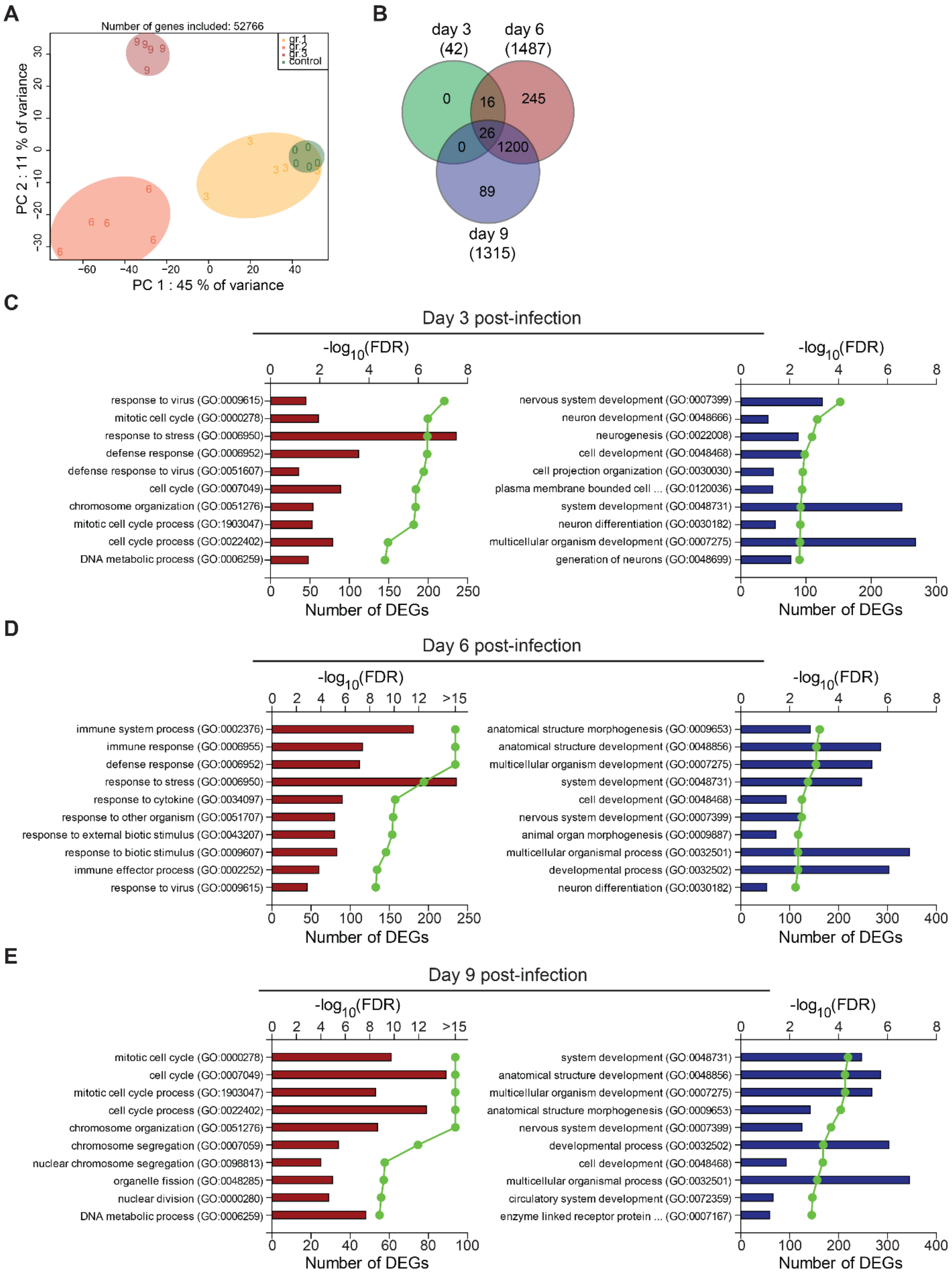
A
Number of genes included: 52766
gr.1
gr.2
gr.3
control
PC 2 : 11 % of variance
PC 1 : 45 % of variance
B
day 3
(42)
day 6
(1487)
day 9
(1315)
0
16
245
26
0
1200
89
C
Day 3 post-infection
-log10(FDR)
Number of DEGs
response to virus (GO:0009615)
mitotic cell cycle (GO:0000278)
response to stress (GO:0006950)
defense response (GO:0006952)
defense response to virus (GO:0051607)
cell cycle (GO:0007049)
chromosome organization (GO:0051276)
mitotic cell cycle process (GO:1903047)
cell cycle process (GO:0022402)
DNA metabolic process (GO:0006259)
nervous system development (GO:0007399)
neuron development (GO:0048666)
neurogenesis (GO:0022008)
cell development (GO:0048468)
cell projection organization (GO:0030030)
plasma membrane bounded cell ... (GO:0120036)
system development (GO:0048731)
neuron differentiation (GO:0030182)
multicellular organism development (GO:0007275)
generation of neurons (GO:0048699)
D
Day 6 post-infection
-log10(FDR)
Number of DEGs
immune system process (GO:0002376)
immune response (GO:0006955)
defense response (GO:0006952)
response to stress (GO:0006950)
response to cytokine (GO:0034097)
response to other organism (GO:0051707)
response to external biotic stimulus (GO:0043207)
response to biotic stimulus (GO:0009607)
immune effector process (GO:0002252)
response to virus (GO:0009615)
anatomical structure morphogenesis (GO:0009653)
anatomical structure development (GO:0048856)
multicellular organism development (GO:0007275)
system development (GO:0048731)
cell development (GO:0048468)
nervous system development (GO:0007399)
animal organ morphogenesis (GO:0009887)
multicellular organismal process (GO:0032501)
developmental process (GO:0032502)
neuron differentiation (GO:0030182)
E
Day 9 post-infection
-log10(FDR)
Number of DEGs
mitotic cell cycle (GO:0000278)
cell cycle (GO:0007049)
mitotic cell cycle process (GO:1903047)
cell cycle process (GO:0022402)
chromosome organization (GO:0051276)
chromosome segregation (GO:0007059)
nuclear chromosome segregation (GO:0098813)
organelle fission (GO:0048285)
nuclear division (GO:0000280)
DNA metabolic process (GO:0006259)
system development (GO:0048731)
anatomical structure development (GO:0048856)
multicellular organism development (GO:0007275)
anatomical structure morphogenesis (GO:0009653)
nervous system development (GO:0007399)
developmental process (GO:0032502)
cell development (GO:0048468)
multicellular organismal process (GO:0032501)
circulatory system development (GO:0072359)
enzyme linked receptor protein ... (GO:0007167)

142

Supplemental Figure 2. Microarray gene expression profiling of lung tissue from SVV-infected cynomolgus macaques**.** (**A**) Principal component analysis of microarray results, with the first and second principal components (PC1, PC2) and their corresponding variances depicted on the *x*- and *y*-axis. Colored numbers indicate day post-infection (dpi). (**B**) Venn diagram showing the number of significant differentially expressed host genes at 3, 6 and 9 dpi (in brackets), and the overlap between each set of differentially expressed genes (total: 50,257 genes analyzed. (**C-E**) Panther Gene Set Enrichment Analysis identified statistically significant (False Discovery Rate (FDR) < 0.05) Gene Ontology (GO) Biological Processes upregulated (red bars) and downregulated (blue bars) at 3 days post-infection (dpi) (**C**), 6 dpi (**D**) and 9 dpi (**E**). Bars indicate number of differentially expressed genes (DEGs) and green line indicates $-\log_{10}$ FDR.

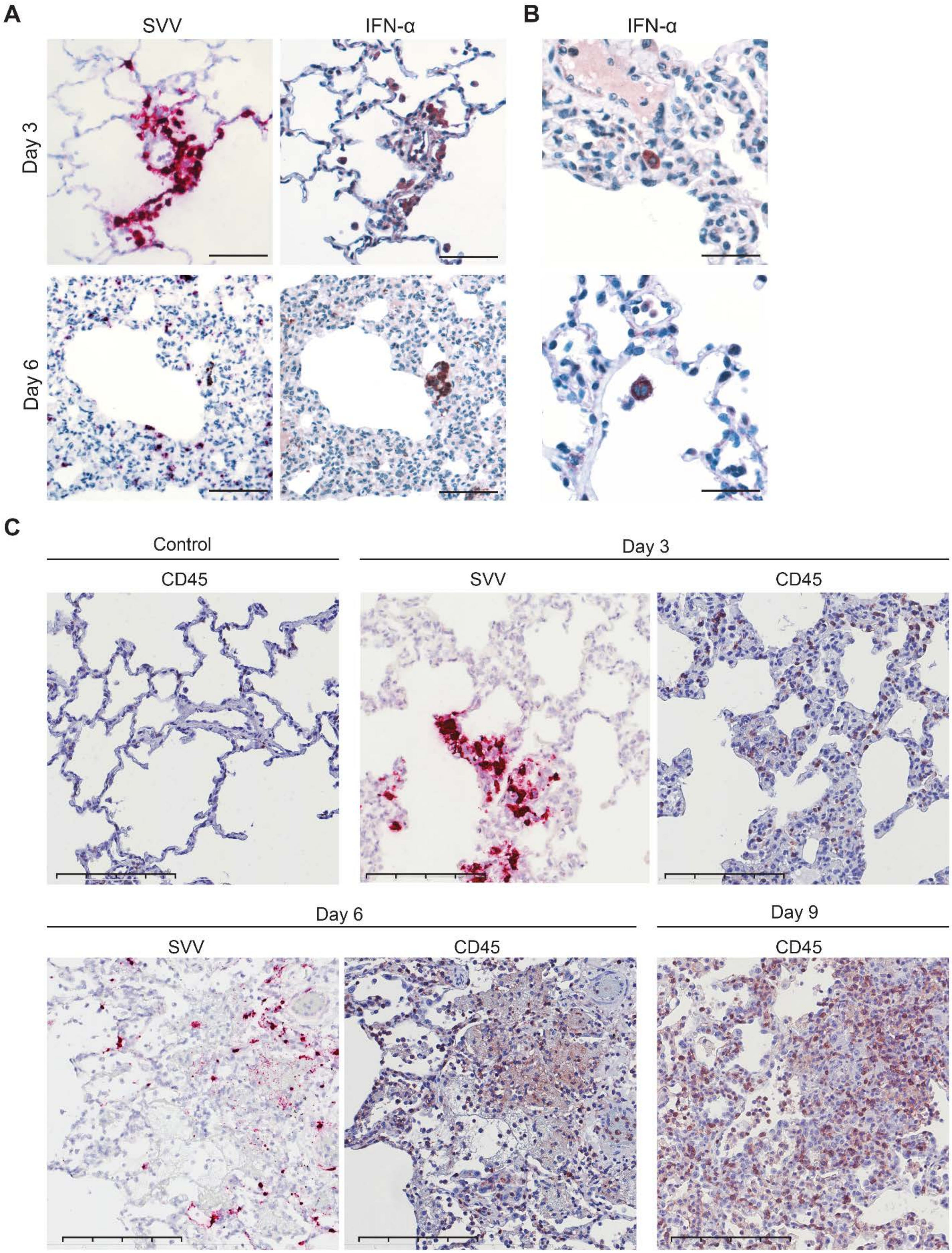
A
SVV
IFN-α
Day 3
Day 6
B
IFN-α
C
Control
CD45
Day 3
SVV
CD45
Day 6
SVV
CD45
Day 9
CD45

Supplemental Figure 3. Detection of innate immune responses and immune cells in lung tissue of SVV-infected cynomolgus macaques. (**A-B**) IFN-α production in lungs of SVV-infected cynomolgus macaques. Consecutive lung sections were analyzed for SVV ORF63 RNA by ISH (red/purple) and IFN-α by IHC (red/brown). Nuclei are stained with hematoxylin (blue). (**A**) IFN-α producing cells were occasionally observed in close proximity to SVV-infected cells at 3 dpi. By contrast, most IFN-α producing cells were located in alveolar inflammatory cell infiltrates associated with SVV ORF63 RNA signal at 6 dpi. (**B**) Low numbers of individual IFN-α producing cells were detected scattered throughout the lung sections of SVV-infected animals at 3 and 6 dpi. (**C**) Co-localization of SVV ORF63 RNA expression and leukocytes in lungs of SVV-infected cynomolgus macaques. Consecutive lung sections were analyzed for SVV ORF63 RNA by ISH (red/pink) and CD45 by IHC (red/brown). Nuclei are stained with hematoxylin (blue). Note that at 3 and 6 dpi the accumulation of $CD45^{pos}$ cells was always associated with the detection of SVV ORF63 in the same area of the adjacent tissue section. Scale bars indicate 100 µm (**A**), 50 µm (**B**) and 200 µm (**C**).

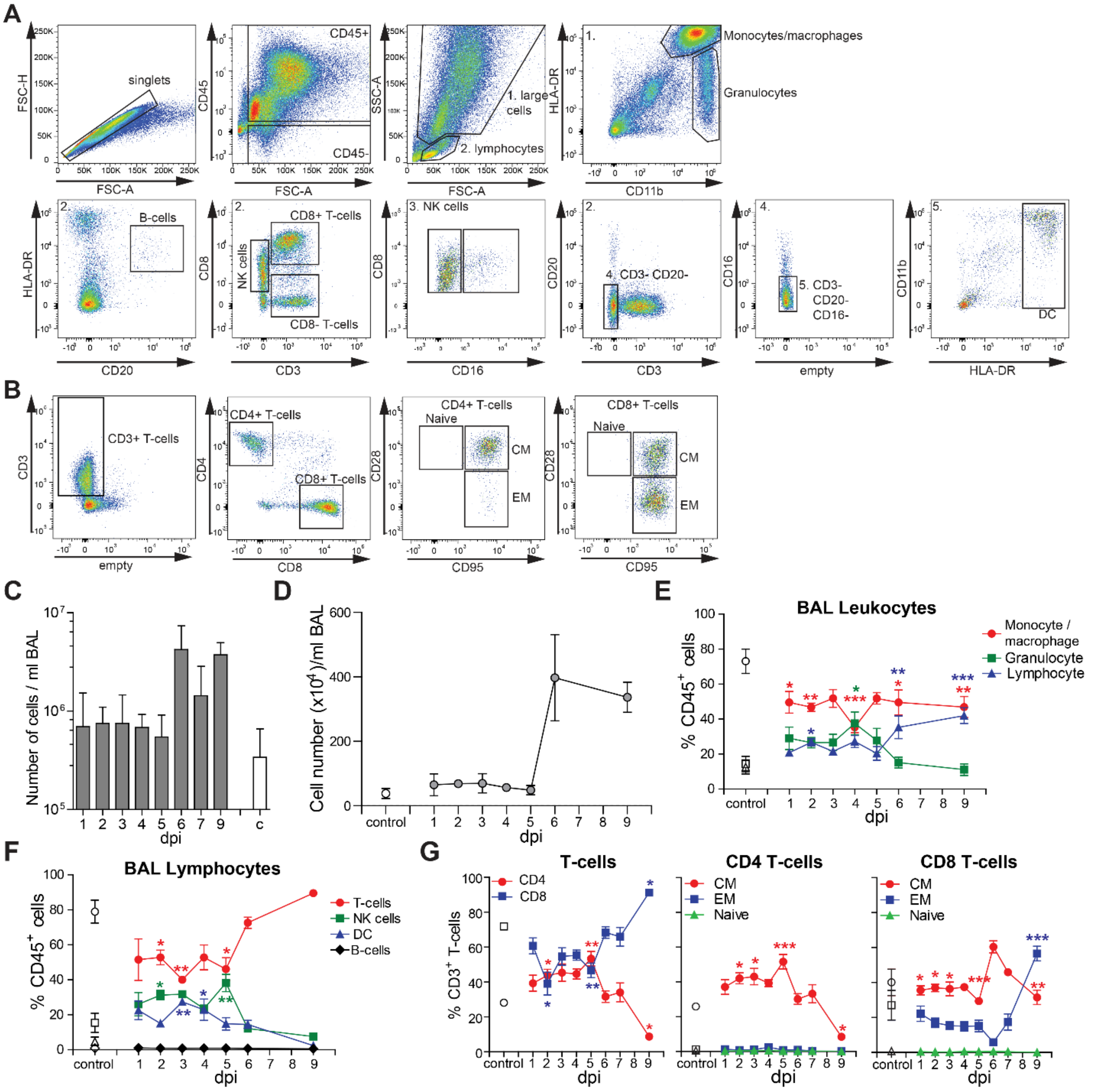

Supplemental Figure 4. Flow cytometric analysis of BAL cells of SVV-infected cynomolgus macaques. Gating strategy for *ex vivo* flow cytometric analysis of BAL samples. (**A**) BAL cells were gated on single, CD45+ cells and divided into large cells and lymphocytes based on FSC-A and SSC-A intensity. Large cells (population #1) were classified as CD11b^{+}HLA-DR^{high} monocytes/macrophages and CD11b^{+}HLA-DR^{dim} granulocytes. Lymphocytes (population #2) were classified as HLA-DR^{+}CD20^{+} B-cells, CD3^{-}CD8^{+} NK cells (population #3) that could be either CD16^{+} or CD16^{-}, CD3^{+} T-cells and CD3^{-}CD20^{-}CD16^{-}HLA-DR^{high}CD11b$^{+/-}$ DC. (**B**) T-cells were further categorized as CD4^{+} T-cells and CD8^{+} T-cells and the respective T-cell subsets naïve

($CD28^+CD95^-$), central memory (CM; $CD28^+CD95^+$) and effector memory (EM; $CD28^-CD95^+$). (**A-B**) Pseudo color density plots are shown for a BAL sample obtained at 1 day post-infection. (**C-D**) Number of viable cells (**C**) and $CD45^+$ cells (**D**) per ml recovered from BAL fluid. (**E-G**) Graphs showing the composition of BAL cell populations (**C**) and T-cell populations (**D**), as determined by flow cytometry. CM: central memory; EM: effector memory. Symbols and error bars indicate mean values ± SEM. * $p < 0.05$, ** $p < 0.01$, *** $p < 0.001$ by one-way ANOVA and Bonferroni correction.

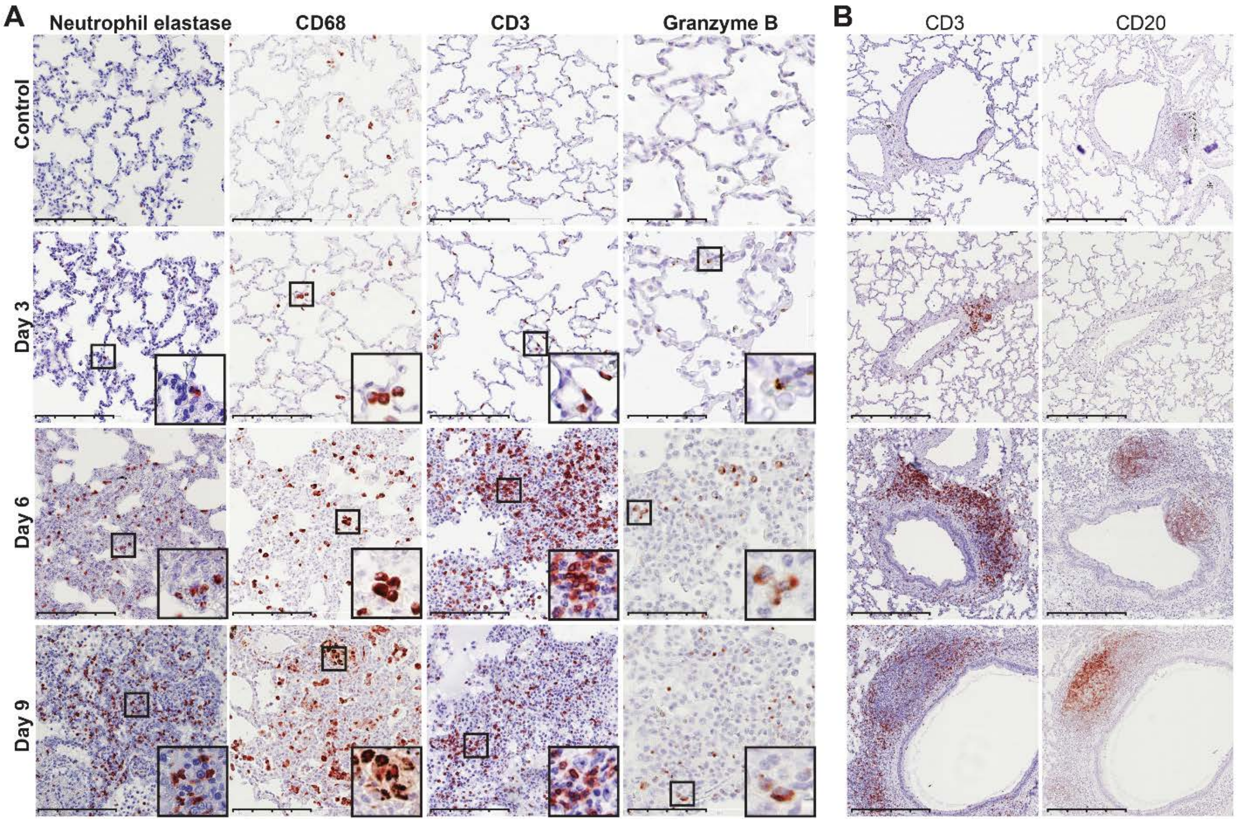

Supplemental Figure 5. Detection of immune cells in lung tissue of SVV-infected cynomolgus macaques. Lung sections from control and SVV-infected animals were analyzed for neutrophil elastase, CD68, CD3 and granzyme B expression in alveoli (**A**) and BALT (**B**) by IHC (red). Sections were counterstained with hematoxylin (blue). Enlargement of areas indicated by black boxes are shown. Scale bars: 100 µm (neutrophil elastase, CD68 and CD3) or 200 µm (granzyme B).

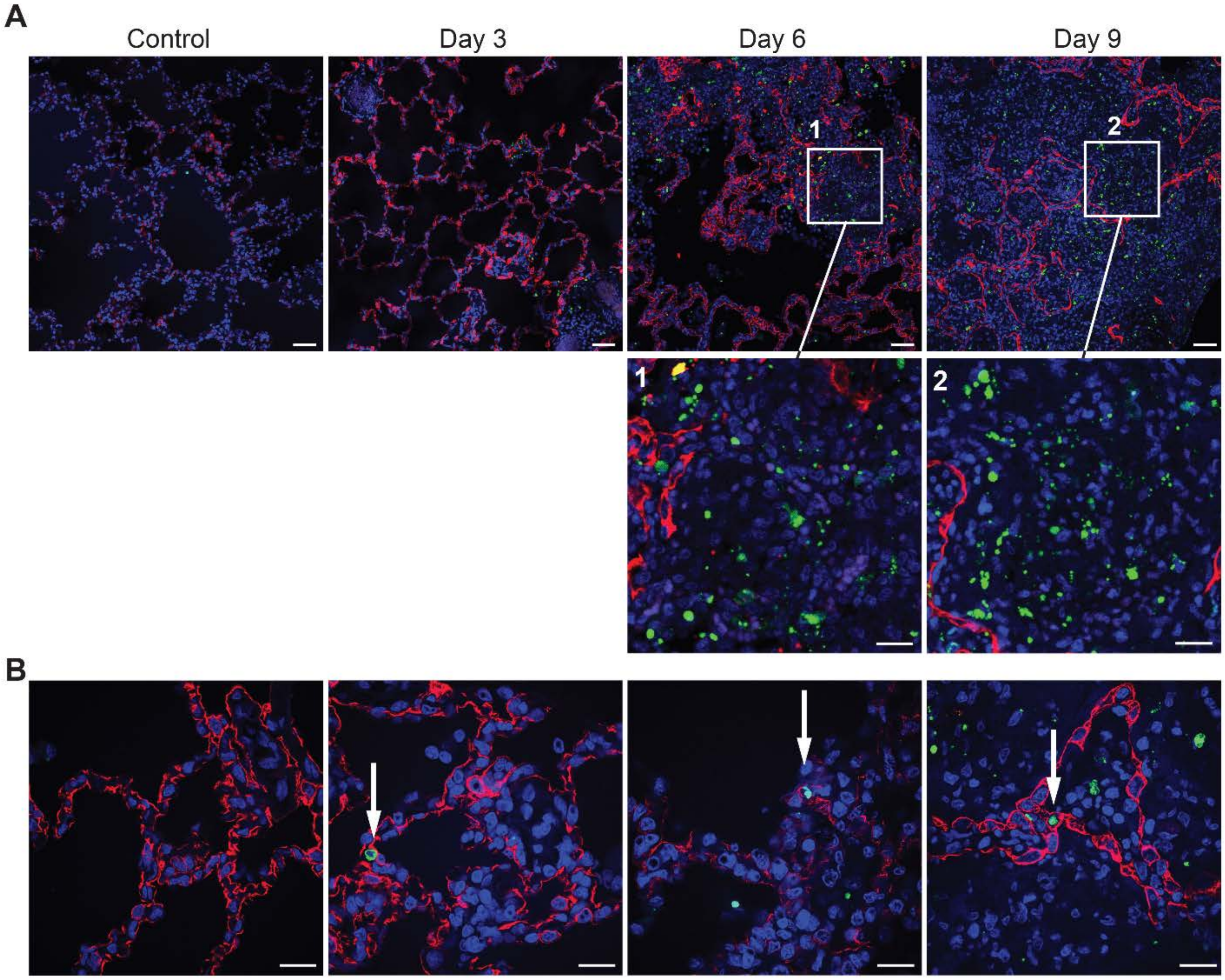

Supplemental Figure 6. Apoptosis in lung tissue of SVV-infected cynomolgus macaques. (**A-B**) Lung sections of mock- and SVV-infected cynomolgus macaques were stained for apoptosis using the TUNEL assay (green) and cytokeratin (red). (**A**) Few apoptotic cells were detected in control animals. A mild increase in apoptotic cells, occasionally identified as alveolar epithelial cells, was observed in SVV-infected animals at 3 dpi. Higher numbers of apoptotic cells were detected in lungs of SVV-infected animals at 6 dpi and 9 dpi. However, majority of TUNEL-positive cells were located within inflammatory cell infiltrates, most likely representing leukocytes, with only incidental detection of apoptotic alveolar epithelial cells (**B**). Enlargements of areas indicated by numbered white boxes in panel A are shown. Nuclei were stained with Hoechst-33342 (blue). Scale bars indicate 50 µm in panel (**A**) and 20 µm in panel (**B**). Arrows indicate TUNEL-positive epithelial cells.

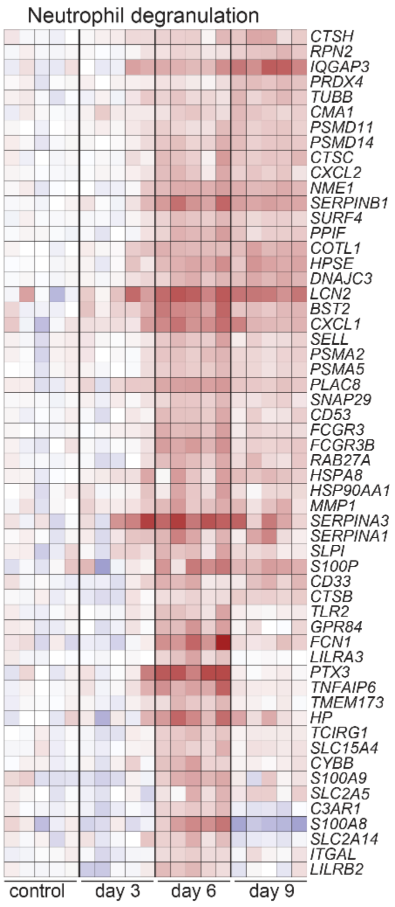

Supplemental Figure 7. Heatmap showing DEGs involved in neutrophil degranulation. Log_2-fold change in gene expression is shown, color gradient: blue = -3, white = 0, red = +6.

Supplemental Table 1. Characteristics of patients with VZV and influenza virus pneumonia.

Patient	Virus	Age (yrs)	Gender	Immunocompetent	Outcome
1	VZV	38	male	Yes	Survived
2	VZV	71	male	No[a]	Died[b]
3	VZV	47	male	Yes	Survived
4	influenza A virus[c]	45	female	Yes	Survived
5	influenza B virus[d]	52	female	Yes	Survived
6	influenza A virus	39	female	Yes	Survived
7	influenza A virus	27	male	Yes	Survived
8	influenza B virus[d]	30	female	Yes	Survived
9	influenza A virus[c]	64	female	Yes	Survived
10	influenza A virus[c]	59	male	Yes	Survived

[a] Rheumatoid arthritis treated with methotrexate, prednisone, azathioprine. [b] Cause of death: respiratory insufficiency. [c] Patient superinfected with *Streptococcus pneumoniae*; [d] Patient superinfected with *Staphylococcus aureus*.

Supplemental Table 2. Primer and probe sequences.

Target	Primer	Sequence	5'-modification	3'-modification
OSM	OSM_F	CCT-CGG-GCT-CAG-GAA-CAA-C	none	none
OSM	OSM_R	GGC-CTT-CGT-GGG-CTC-AG	none	none
OSM	OSM_P	TAC-TGC-ATG-GCC-CAG-CTG-CTG-GAC-AA	FAM	BHQ-1
SVV ORF21	ORF21_F	GACACATCAGCGGTTTGCA	none	none
SVV ORF21	ORF21_R	TGCACGCTGTGTTAGAATTCG	none	none
SVV ORF21	ORF21_P	TCCATCCTGAACGATAGGCATGTCATAAAGA	FAM	BHQ-1
SVV ORF63	ORF63_F	CGTACGCTCCGCTGACAAA	none	none
SVV ORF63	ORF63_R	TGCTGTCCAATGCGTTTCTG	none	none
SVV ORF63	ORF63_P	CGTCCCCGCACAATTACAGCGC	FAM	BHQ-1
